# Integrating the impact of global change on the niche and physiology of marine nitrogen‐fixing cyanobacteria

**DOI:** 10.1111/gcb.16399

**Published:** 2022-09-13

**Authors:** Lewis Wrightson, Nina Yang, Claire Mahaffey, David A. Hutchins, Alessandro Tagliabue

**Affiliations:** ^1^ Department of Earth, Ocean and Ecological Sciences University of Liverpool Liverpool UK; ^2^ Department of Biological Sciences University of Southern California Los Angeles California USA

**Keywords:** climate change, earth system models, elemental use efficiency, marine nitrogen fixation, microbial thermal fitness

## Abstract

Marine nitrogen fixation is a major source of new nitrogen to the ocean, which interacts with climate driven changes to physical nutrient supply to regulate the response of ocean primary production in the oligotrophic tropical ocean. Warming and changes in nutrient supply may alter the ecological niche of nitrogen‐fixing organisms, or ‘diazotrophs’, however, impacts of warming on diazotroph physiology may also be important. Lab‐based studies reveal that warming increases the nitrogen fixation‐specific elemental use efficiency (EUE) of two prevalent marine diazotrophs, *Crocosphaera* and *Trichodesmium*, thus reducing their requirements for the limiting nutrients iron and phosphorus. Here, we coupled a new diazotroph model based upon observed diazotroph energetics of growth and resource limitation to a state‐of‐the‐art global model of phytoplankton physiology and ocean biogeochemistry. Our model is able to address the integrated response of nitrogen fixation by *Trichodesmium* and *Crocosphaera* to warming under the IPCC high emission RCP8.5 scenario for the first time. Our results project a global decline in nitrogen fixation over the coming century. However, the regional response of nitrogen fixation to climate change is modulated by the diazotroph‐specific thermal performance curves and EUE, particularly in the Pacific Ocean, which shapes global trends. Spatially, the response of both diazotrophs is similar with expansion towards higher latitudes and reduced rates of nitrogen fixation in the lower latitudes. Overall, 95%–97% of the nitrogen fixation climate signal can be attributed to the combined effect of temperature on the niche and physiology of marine diazotrophs, with decreases being associated with a reduced niche and increases resulting due to a combination of expanding niche and temperature driven changes to EUE. Climate change impacts on both the niche and physiology of marine diazotrophs interact to shape patterns of marine nitrogen fixation, which will have important implications for ocean productivity in the future.

## Graphical abstract

To explore the impact of global change on marine nitrogen fixation, thermal performance curves of growth and resource limitation for two prevalent marine nitrogen‐fixing cyanobacteria were incorporated into a state‐of‐the‐art Earth System Model for the first time. Marine nitrogen fixation is predicted to decrease globally over the coming century, with the combined effect of temperature on the niche and physiology of marine diazotrophs explaining 95%–97% of the nitrogen fixation climate signal. Climate change impacts on both the niche and physiology on marine diazotrophs interact to shape patterns of marine nitrogen fixation, with important implications for ocean productivity in the future.

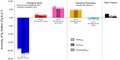

## 1 INTRODUCTION

Marine dinitrogen (N_2_) fixation, or ‘diazotrophy’ is a key source of reactive nitrogen (N) to the global ocean supplying between 68 and 164 Tg N year^−1^ (Gruber & Sarmiento, 1997; Jickells et al., 2017; Luo et al., 2014; Tang et al., 2019; Wang et al., 2019) and fuels primary production in N limited regions of the ocean. Earth system models (ESM) project that N_2_ fixation will decline over the coming century. As the climate driven signal in marine N_2_ fixation emerges earlier than the trends in primary productivity, marine N_2_ fixation may shape the response of primary producers to climate change (Wrightson & Tagliabue, 2020). The predicted increase in ocean temperature will affect multiple aspects of diazotrophy, with emphasis to date on the impact of warming on stratification and nutrient supply (Luo et al., 2014; Sohm et al., 2011; Weber & Deutsch, 2014), with some work on how changing temperature will alter the physiology and thermal niche of diazotrophs (Fu et al., 2014; Jiang et al., 2018; Yang et al., 2021). Increasing sea surface temperature (SST) plays a primary role in controlling the thermal niche of diazotrophs. In the low latitudes, warming may surpass their thermal maximum leading to exclusion, whilst increasing temperatures below their thermal maximum allows poleward expansion (Boatman et al., 2020; Breitbarth et al., 2007; Fu et al., 2014). Changing nutrient supply due to stratification can either open a competitive niche for diazotrophy if the supply of N declines such that it limits non‐diazotrophs (Weber & Deutsch, 2010), or it can restrict rates of N_2_ fixation if the supply of phosphorus (P) or iron (Fe) declines (Hutchins & Capone, 2022).

Alongside temperature and nutrient availability, another potentially important driver that may impact marine diazotrophy is carbon dioxide (CO_2_). When *Trichodesmium* and *Crocosphaera* are exposed to increased concentrations of CO_2_, enhanced growth and N_2_ fixation rates have been observed, and it has been suggested that like temperature, CO_2_ may define an upper limit on N_2_ fixation rates (Hutchins et al., 2007, 2013, 2015; Walworth et al., 2021). Increased CO_2_ concentrations have been proposed to reduce the diazotroph's requirement for carbon concentrating mechanisms (CCM), enabling more energetic investment into N_2_ fixation, photosynthesis, and growth (Boatman et al., 2018). However, CO_2_ only has a strong impact on diazotrophy under Fe replete conditions (Fu et al., 2008; Walworth et al., 2016). These results imply that increasing CO_2_ in the future may benefit marine diazotrophs mostly in regions that are replete in Fe, such as the tropical North Atlantic Ocean.

**FIGURE 1:**
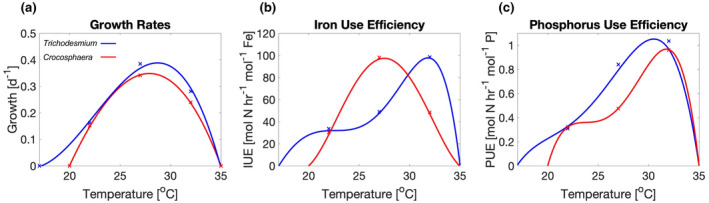
Thermal performance curves of growth (a), iron use efficiency (b), and phosphorus use efficiency (c) for *Trichodesmium* (blue) and *Crocosphaera* (red). Curves were fitted to the data from Jiang et al. (2018) for *Trichodesmium* and from Yang et al. (2021) for *Crocosphaera*. Data points are shown by crosses.

Temperature can also indirectly impact diazotroph growth by influencing enzyme efficiency and altering diazotroph physiology. Recent studies have used the concept of elemental use efficiencies (EUE) to account for the effect of temperature on enzyme efficiency and resource requirements of diazotrophy in an integrated manner (Jiang et al., 2018; Yang et al., 2021). Thermal shifts in N_2_ fixation specific EUEs are calculated by measuring the rate of N_2_ fixation normalized to the cellular element quotas of the diazotroph (e.g., using the Fe quota gives the iron use efficiency [IUE]), and observing how it changes across the diazotroph's thermal window. An increase in the EUE means that the diazotroph is performing more N_2_ fixation per unit element considered, leading to a reduction in the nutrient demand of the diazotroph. These temperature driven changes to diazotroph physiology are mediated by changes in the biological utilization of the limiting nutrients Fe and P in response to warming. Thermal performance curves and N_2_ fixation specific EUEs for P and Fe have been measured for two marine diazotrophs, *Trichodesmium* and *Crocosphaera*. *Crocosphaera* has a narrower thermal window for growth than *Trichodesmium*, as it grows between 20 and 35°C compared to 17 and 35°C for *Trichodesmium* (Boyd et al., 2013) with the thermal optimum for growth occurring at 28.7 and 27.9°C for *Trichodesmium* and *Crocosphaera*, respectively (Figure 1a; Jiang et al., 2018; Yang et al., 2021). The N_2_ fixation EUEs also respond differently to temperature depending on the element and the diazotroph in question. The thermal optimum for *Trichodesmium* IUE and phosphorus use efficiency (PUE) occur at 31.8 and 30.5°C, respectively, while for *Crocosphaera* IUE and PUE the thermal optimums occur at 27.5 and 31.8°C, respectively (Figure 1b,c; Jiang et al., 2018; Yang et al., 2021). The different responses of both diazotrophs to temperature, including their growth rates, iron, and phosphorus use efficiencies, highlight the need for more information on how thermal fitness of each diazotroph shapes the response of diazotrophy to future ocean warming.

ESMs are the main tool to investigate how the future ocean will respond to climate change, and their results underpin important assessments by the IPCC (Eyring et al., 2016; van den Hurk et al., 2018). However, current ESMs have an incomplete representation of N_2_ fixation as focus is primarily upon the impacts of temperature on the niche of marine diazotrophs (Wrightson & Tagliabue, 2020). As temperature has the potential to modulate not only the extent of the thermal niche of diazotrophs but also their physiology via changing EUEs, diazotroph thermal fitness dynamics need to be incorporated into ESMs to assess the integrated climate change response (Boatman et al., 2020; Jiang et al., 2018; Yang et al., 2021). Such models should account for the temperature impacts on both the growth and niche of diazotrophs, as well as incorporating the effects of warming on diazotroph physiology via EUEs. Alongside these factors, changes in the physical environment (driven by warming, but also by changes in winds and salinity) will also alter the availability of nutrients. As growth rates and EUEs respond to temperature distinctly between diazotrophs, there is also a need to assess whether the ESM parameterizations based on *Trichodesmium* or *Crocosphaera* affect the response of diazotrophy to changes in climate. To date, the effects of temperature on growth and IUE for *Crocosphaera* and *Trichodesmium* have been assessed using an additive Michaelis–Menten based approach in response to annual average Fe concentration and SST from the NCAR CMIP5 model under the high emissions RCP8.5 scenario. The diagnostic modelling results suggest that N_2_ fixation rates will increase globally by 22% and 91% for *Trichodesmium* and *Crocosphaera*, respectively (between two time slices at 2010 and 2100) due to increased IUEs and expansion of the diazotroph niche (Jiang et al., 2018; Yang et al., 2021). However, these diagnostic models focused on only temperature and Fe limitation, neglecting the role of other bottom‐up and top‐down drivers such as P limitation, light limitation, grazing, and competition with other phytoplankton in a fully prognostic sense. The susceptibility of diazotrophs to Fe limitation also varies as diazotrophs deploy different N_2_ fixation strategies that can affect their Fe demand. For example, *Trichodesmium* performs N_2_ fixation and photosynthesis simultaneously during the day, whilst *Crocosphaera* temporally segregates both processes by performing photosynthesis during the day and N_2_ fixation at night (Berman‐Frank et al., 2007). *Trichodesmium* is therefore required to satisfy the Fe demand of both processes simultaneously, while *Crocosphaera* can deploy a ‘hot bunking’ strategy that cycles the same cellular Fe pool between the two processes over the diel cycle. This has been suggested to reduce the Fe cost of *Crocosphaera* by 40%–50% compared to that required by *Trichodesmium* to fix the same amount of N_2_ (Saito et al., 2011). Diazotrophs respond not only to temperature and Fe availability but to a suite of drivers such as grazing, light limitation, and fixed N, which can affect growth rates and alter the niche of diazotrophy. To assess the impact of climate change on marine diazotrophy, a holistic consideration of how temperature can affect diazotroph thermal fitness and N_2_ fixation rates in the future is required (Hutchins & Capone, 2022).

The aim of this study was to investigate how diazotroph thermal fitness, both in terms of a changing thermal niche and EUEs, responds to climate change under the high emissions RCP8.5 scenario. To do this, we developed a new state‐of‐the‐art diazotroph compartment for the PISCES QUOTA model based upon observed diazotroph thermal performance curves of growth and EUEs to account for the thermal fitness of two marine diazotrophs, *Trichodesmium* and *Crocosphaera*, which are interchangeable within the model. Here, we describe the new model and experiments focused on investigating how the response of N_2_ fixation to climate change differs between *Trichodesmium* and *Crocosphaera*, at regional scales.

## 2 MODEL DESCRIPTION

The new diazotroph model was developed for the PISCES QUOTA ESM, which allows for complete variable phytoplankton stoichiometry and applies optimal allocation of resources (Kwiatkowski et al., 2018). In the model, diazotroph growth and N_2_ fixation are limited by temperature, light, and nutrient availability (P and Fe). N_2_ fixation is facultative, allowing the diazotroph to use other forms of fixed N (nitrate and ammonium) (Holl & Montoya, 2005; Knapp, 2012; Mulholland et al., 2001). That said, diazotroph maximum growth rates are much lower than those ascribed to diatoms, nanophytoplankton and picoplankton, which results in their exclusion when only nitrate and ammonia are used as a N source. The full model description can be found in the supplementary material. Within the model, diazotroph nutrient requirements are set by the prescribed minimum quotas, which restrict growth when nutrient concentrations do not satisfy the minimum quota. For N and P, the minimum quota is allometrically scaled, but the initial value of the minimum N and P quotas are predefined. For Fe however, the minimum quota ($Q_{\mathrm{Fe},\min}^{\mathrm{dz}}$) is variable and is calculated as the sum of Fe costs for photosynthesis, respiration, nitrate reductase, and N_2_ fixation (Equation 1),
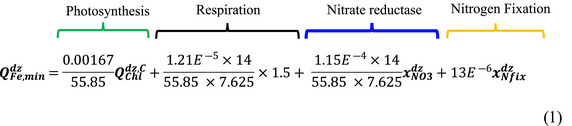


where $Q_{\mathrm{Chl}}^{\mathrm{dz},C}$ = Chlorophyll *α* to C ratio,

(2) $$x_{\text{Nfix}}^{\mathrm{dz}}=1-\left(x_{{\mathrm{NO}}_{3}}^{\mathrm{dz}}+x_{{\mathrm{NH}}_{4}}^{\mathrm{dz}}\right)\;\text{Facultative term}\;\left(\text{proportion of}\;N\;\text{supply from}\;N_{2}\;\text{fixation}\right),$$

where $x_{{\mathrm{NO}}_{3}}^{\mathrm{dz}}$ = proportion of uptake of NO_3_; $x_{{\mathrm{NH}}_{4}}^{\mathrm{dz}}$ = proportion of uptake of NH_4_.

The Fe costs of photosynthesis, respiration, and nitrate reductase used in Equation (1) are taken from Flynn and Hipkin (1999) and follow the approach used for the other phytoplankton functional types (PFT) in PISCES QUOTA with an additional term for diazotrophs to account for the cost of N_2_ fixation (Kwiatkowski et al., 2018). The $x_{\text{Nfix}}^{\mathrm{dz}}$ term represents the proportion of the diazotroph fixed N demand that comes from N_2_ fixation. The Fe cost of N_2_ fixation is based upon the work of Kustka, Sañudo‐Wilhelmy, Carpenter, Capone, and Raven (2003), which suggested that the additional Fe requirement for growth by *Trichodesmium* using N_2_ is ~30–50 × 10^−6^ mol Fe mol^−1^ C, of which nitrogenase, the enzyme required for N_2_ fixation, accounts for ~25%. This implies that the cost of nitrogenase is ~10 × 10^−6^ mol Fe mol^−1^ C. Diazotrophs also rely on the Mehler reaction which produces free oxygen radicals. In order to consume these free oxygen radicals, diazotrophs employ superoxide dismutase, which has an Fe cost of ~3 × 10^−6^ mol Fe mol^−1^ C. The overall Fe cost for satisfying all the diazotrophs N demand from N_2_ fixation is therefore 13 × 10^−6^ mol Fe mol^−1^ C.

For this new version of the diazotroph model, the diazotroph PFT can switch between a *Trichodesmium* and *Crocosphaera* parameterization, which then alters the thermal performance curves of growth and EUEs appropriately (Figure 1). The growth curves used in the model (Equation 3) were obtained by fitting a curve to observations of *Trichodesmium* (Jiang et al., 2018) and *Crocosphaera* (Yang et al., 2021) growth rates over a range of temperatures. Observations of EUEs were also obtained and had curves fitted to produce the thermal performance curves for both Fe and P EUEs (Figure 1). As the EUEs increase, the nutrient demand should decrease. Therefore, the IUE curve was then used as a simple scalar for the Fe cost of N_2_ fixation. Similarly, the PUE curve was used as a scalar for the minimum P quota of the diazotroph. In this way, when the EUEs increased, the cellular Fe or P requirements decreased and when the EUEs decreased, the cellular Fe and P requirements increased. The EUEs used in this study were derived from experiments conducted under replete nutrient conditions (Jiang et al., 2018; Yang et al., 2021) to better isolate the direct and indirect drivers. The ensuing EUEs that emerge from the model integrate the effect of nutrient limitation. We used the observed thermal response curves for growth to set the maximum growth rate of each diazotroph, which is then controlled by temperature, light, and nutrient availability. This model was then run using either a fixed or temperature sensitive EUE for comparison. As our model only represents a single diazotroph for each experiment, it cannot account for any direct competition between both diazotrophs at this time. However, our model is able to highlight how different diazotroph assumptions influence the model responses to spatial and temporal variability. We model the thermal performance curve for diazotroph maximum growth rates via the following generic empirical equation:

(3) $$\mu_{\max}^{\mathrm{dz}}=a_{\mathrm{dz}}^{\mu }T^{3}+b_{\mathrm{dz}}^{\mu }T^{2}+c_{\mathrm{dz}}^{\mu }T+d_{\mathrm{dz}}^{\mu },$$

where $\mu_{\max}^{\mathrm{dz}}$ = maximum diazotroph growth rate (day^−1^); T = temperature (°C).

The temperature range for *Trichodesmium* growth was set from 17 to 35°C, while *Crocosphaera* has a narrower thermal window with growth permitted between 20 and 35°C. Values used to calculate the growth curves for both diazotrophs in Equation (3) are shown in Table 1.

**TABLE 1:**
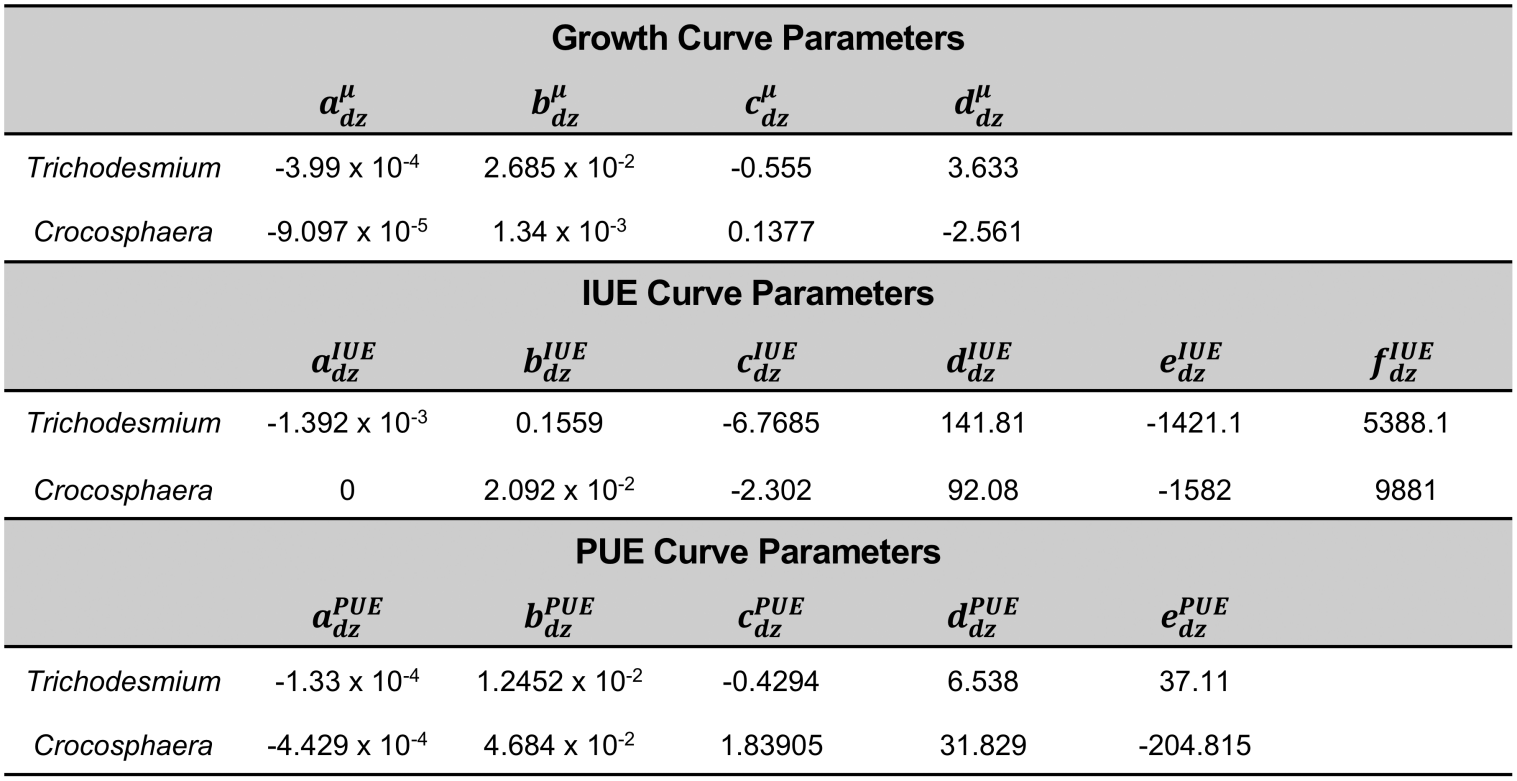
Values used to calculate the thermal performance curves for growth and elemental use efficiencies based on observations of *Trichodesmium* and C*rocosphaera* (Figure 1)

### 2.1 Calculation of the nitrogen fixation EUEs for both diazotrophs

To incorporate the thermal performance curves of the EUEs into the model, we fitted a curve to the observations from Jiang et al. (2018) and Yang et al. (2021) (Figure 1b,c; Equations 4 and 5) and converted them into a scaling term where the scaling was set to 1 when the diazotroph growth rate was 0.1 day^−1^, which was the reference growth rate for the calculation of the Fe cost of N_2_ fixation (Kustka, Sañudo‐Wilhelmy, Carpenter, Capone, & Raven, 2003). At a growth rate of 0.1 day^−1^, the Fe cost of N_2_ fixation is 13 × 10^−6^ mol Fe mol^−1^ C, and, since the EUE modulates the nutrient demand, the IUE scaling relationship was then used to scale the Fe cost of N_2_ fixation, and the PUE scaling relationship was used to scale the minimum P quota. The generic scaling equations are

(4) $${\mathrm{IUE}}^{\mathrm{dz}}=a_{\mathrm{dz}}^{\mathrm{IUE}}T^{5}+b_{\mathrm{dz}}^{\mathrm{IUE}}T^{4}+c_{\mathrm{dz}}^{\mathrm{IUE}}T^{3}+d_{\mathrm{dz}}^{\mathrm{IUE}}T^{2}+e_{\mathrm{dz}}^{\mathrm{IUE}}T+f_{\mathrm{dz}}^{\mathrm{IUE}},$$

(5) $${\mathrm{PUE}}^{\mathrm{dz}}=a_{\mathrm{dz}}^{\mathrm{PUE}}T^{4}+b_{\mathrm{dz}}^{\mathrm{PUE}}T^{3}+c_{\mathrm{dz}}^{\mathrm{PUE}}T^{2}+d_{\mathrm{dz}}^{\mathrm{PUE}}T+e_{\mathrm{dz}}^{\mathrm{PUE}}.$$

Values used to calculate the thermal performance curves of the EUEs of both diazotrophs are provided in Table 1. Within the model, the minimum Fe quota of the diazotroph is set by the sum of several Fe costs (Equation 1). To incorporate the IUEs of N_2_ fixation into the model, a scaling approach was used. Following Kustka, Sañudo‐Wilhelmy, Carpenter, Capone, and Raven (2003), the Fe cost of N_2_ fixation is 13 × 10^−6^ mol Fe mol^−1^ C for a 0.1 day^−1^ growth rate, so the scaling needs to be set to 1 where growth is equal to 0.1 day^−1^, as this was the growth rate at which the reference Fe cost of N_2_ fixation was calculated. The IUE curve was divided by the IUE of the diazotroph when growth was 0.1 day^−1^ (Equation 6). When the IUE increases, the Fe cost of N_2_ fixation would decrease so the reciprocal of the IUE scaling was required (Equation 7). The Fe cost scaling was then used to modulate the Fe cost of N_2_ fixation depending on temperature (Equation 8). Following the approach used for the IUEs, the PUE scaling was performed in a similar manner (Equations 9 and 10). However, to account for the change in the P demand of the diazotroph, the PUE scaling was used to modulate the minimum P quota of the diazotroph (Equation 11),

(6) $$\mathrm{IUE}\;{\text{scaling}}^{\mathrm{dz}}=\frac{{\mathrm{IUE}}^{\mathrm{dz}}}{{\mathrm{IUE}}_{\mu 0.1}^{\mathrm{dz}}},$$

(7) $$\mathrm{Fe}\;{\text{cost scaling}}^{\mathrm{dz}}=\frac{1}{\mathrm{IUE}\;{\text{scaling}}^{\mathrm{dz}}},$$

(8) $$\mathrm{Fe}\;\text{cost of}\;N_{2}\;{\text{fixation}}^{\mathrm{dz}}=13E^{-6}\times x_{\text{Nfix}}^{\mathrm{dz}}\times \mathrm{Fe}\;{\text{cost scaling}}^{\mathrm{dz}},$$

${\mathrm{IUE}}_{\mu 0.1}^{\mathrm{dz}}$ = IUE at 0.1 day^−1^ growth rate (*Trichodesmium* = 33.49 mol N h^−1^ mol^−1^ Fe, *Crocosphaera* = 20.64 mol N h^−1^ mol^−1^ Fe),

(9) $$\mathrm{PUE}\;{\text{scaling}}^{\mathrm{dz}}=\frac{{\mathrm{PUE}}^{\mathrm{dz}}}{{\mathrm{PUE}}_{\mu 0.1}^{\mathrm{dz}}},$$

(10) $${\mathrm{QP}}_{\min}^{\mathrm{dz}}\text{scaling}=\frac{1}{\mathrm{PUE}\;{\text{scaling}}^{\mathrm{dz}}},$$

(11) $${\mathrm{QP}}_{\min}^{\mathrm{dz}}={\mathrm{QP}}_{\min}^{\mathrm{dz}}\times {\mathrm{QP}}_{\min}^{\mathrm{dz}}\text{scaling},$$

${\mathrm{PUE}}_{\mu 0.1}^{\mathrm{dz}}$ = PUE at 0.1 day^−1^ growth rate (*Trichodesmium* = 0.25 mol N h^−1^ mol^−1^ P, *Crocosphaera* = 0.2628 mol N h^−1^ mol^−1^ P).

### 2.2 Model experiments

Several simulations were performed to investigate how climate change affects the different diazotrophs. Our reference simulations include specific thermal performance curves and temperature dependent EUEs for either *Trichodesmium* or *Crocosphaera*. To test for the influence of a lower Fe cost of N_2_ fixation for *Crocosphaera*, we also conducted an additional experiment where the Fe cost of N_2_ fixation was reduced by 40% (7.8 × 10^−6^ mol Fe mol^−1^ C) following Saito et al. (2011). We then conducted a parallel suite of experiments with the temperature dependent EUEs switched off. For each simulation the model was run using forcing from the picontrol simulation from 1801 to 2100, under historical forcing from 1852 to 2005 and under the RCP 8.5 scenario from 2005 to 2100. Reference time periods for the analysis were 1996–2005 for the contemporary state and 2091–2100 for the end of century. The model code can be found https://github.com/lewiswrightson/PISCES‐QUOTA‐P6Z and the output is available on Zenodo (Wrightson et al., 2022).

### 2.3 Model nutrient limitation

**FIGURE 2:**
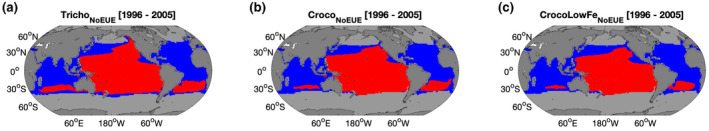
Spatial distribution of the dominant underlying nutrient limitation regimes experienced by the diazotroph phytoplankton functional type with no elemental use efficiencies within the model: (a) *Trichodesmium*, (b) *Crocosphaera*, and (c) *Crocosphaera* with reduced Fe cost of nitrogen fixation. Red regions indicate Fe limitation and blue indicates macronutrient limitation (N or P).

Before discussing the results, it is important to highlight that within the model, strong underlying nutrient limitation regimes are experienced by the diazotrophs between different ocean basins. In the Atlantic and Indian oceans, the diazotrophs within the model are mainly P‐limited with patches of Fe limitation to the South. However, in the Pacific Ocean the dominant limiting nutrient is Fe with P limitation in the North‐West (Figure 2). These nutrient limitation regimes present within the model agree with observations and previous modelling approaches of diazotroph nutrient limitation (Dutkiewicz et al., 2012, 2014; Sohm et al., 2011; Zehr & Capone, 2020). In the Atlantic Ocean, episodic Fe input controls patterns of N_2_ fixation with increased Fe concentrations driving diazotrophs towards P limitation in the North, whilst reduced Fe supply and excess P drive diazotrophs towards Fe limitation in the South (Moore et al., 2009). However, recent proteomics and transcriptomic studies on *Trichodesmium* revealed that throughout the North Atlantic, simultaneous Fe and P limitation, or Fe and P co‐limitation may be more prevalent as opposed to either Fe or P single nutrient limitation (Cerdan‐Garcia et al., 2021; Held et al., 2020). In the Pacific Ocean, molecular evidence suggests that low Fe concentrations result in widespread Fe limitation throughout the basin (Chappell et al., 2012), which has also been corroborated by observations in the North Pacific (Sohm et al., 2008). The nutrient limitation regimes within the model broadly agree with the observations indicating that the model suitably represents overall patterns of diazotroph nutrient limitation. Accounting for the lower Fe cost of N_2_ fixation for *Crocosphaera* reduces the extent of the Fe limited regions by up to 4% (Figure 2c).

## 3 RESULTS AND DISCUSSION

We first focus on the reference simulations for both *Trichodesmium* and *Crocosphaera*, using the state‐of‐the‐art version of the model with both temperature dependent EUEs active (Tricho_REF,_ Croco_REF_, Table 2).

**TABLE 2:**
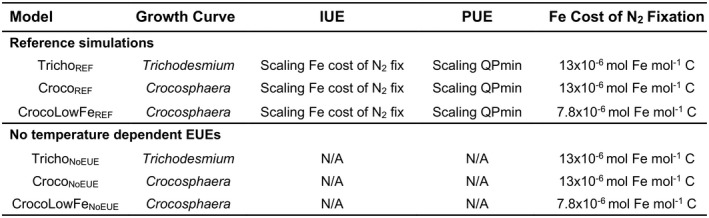
Description of model experiments performed to explore the impact of warming on marine diazotrophy

### 3.1 Regional response of Diazotrophy to climate change

**FIGURE 3:**
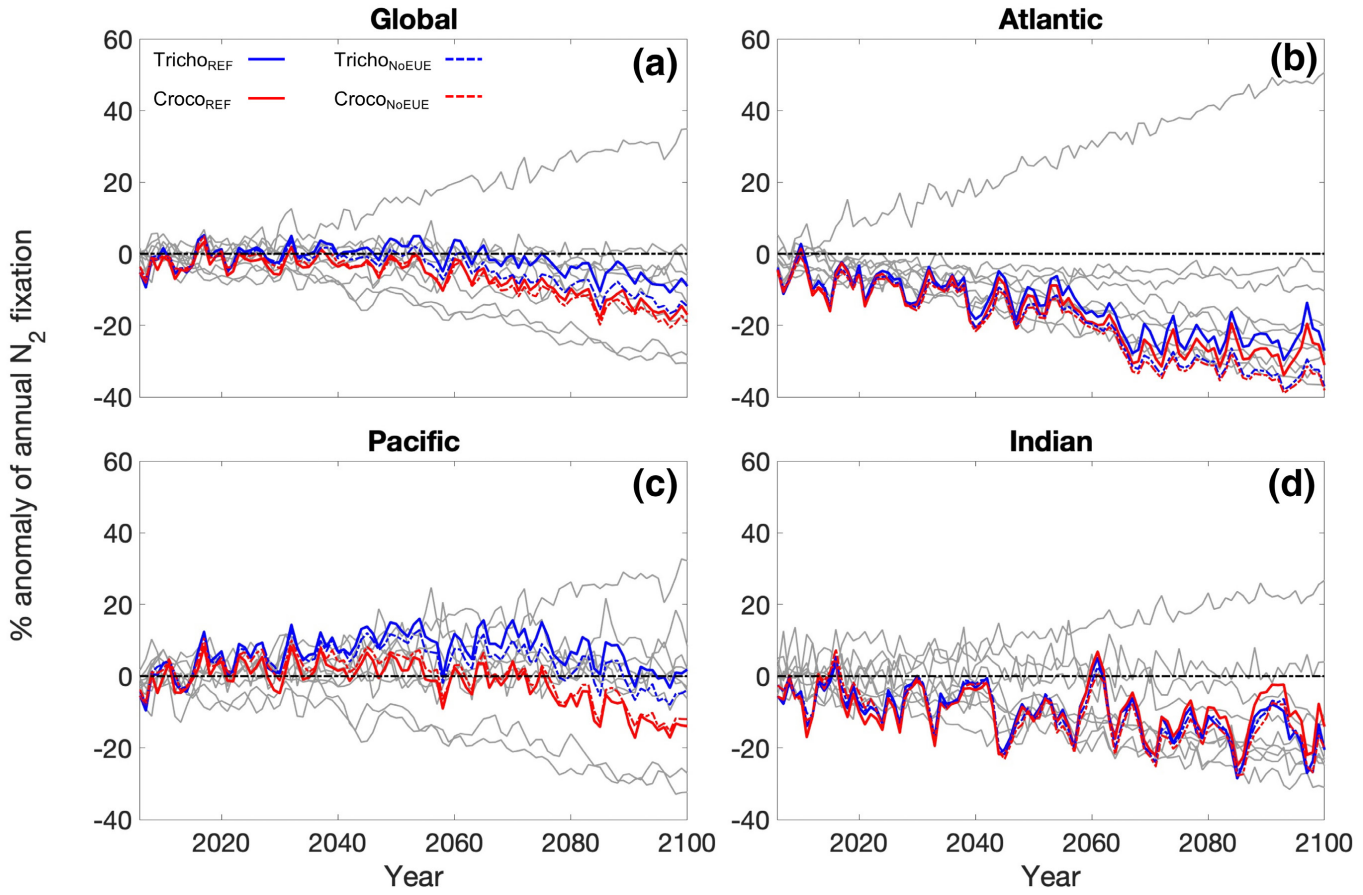
Percentage change of integrated nitrogen fixation for the RCP8.5 scenario (2006–2100) compared to the historical mean (1996–2005) for *Trichodesmium* (blue) and *Crocosphaera* (red), solid lines represent reference simulation where temperature dependent elemental use efficiencies (EUEs) were included, and dashed lines represent model without EUEs. Percentage change is shown for the global ocean (a) and the ocean basins: (b) Atlantic, (c) Pacific and (d) Indian oceans. Grey lines represent 9 ESMs that have been used for climate change projections of nitrogen fixation (Wrightson & Tagliabue, 2020).

**FIGURE 4:**
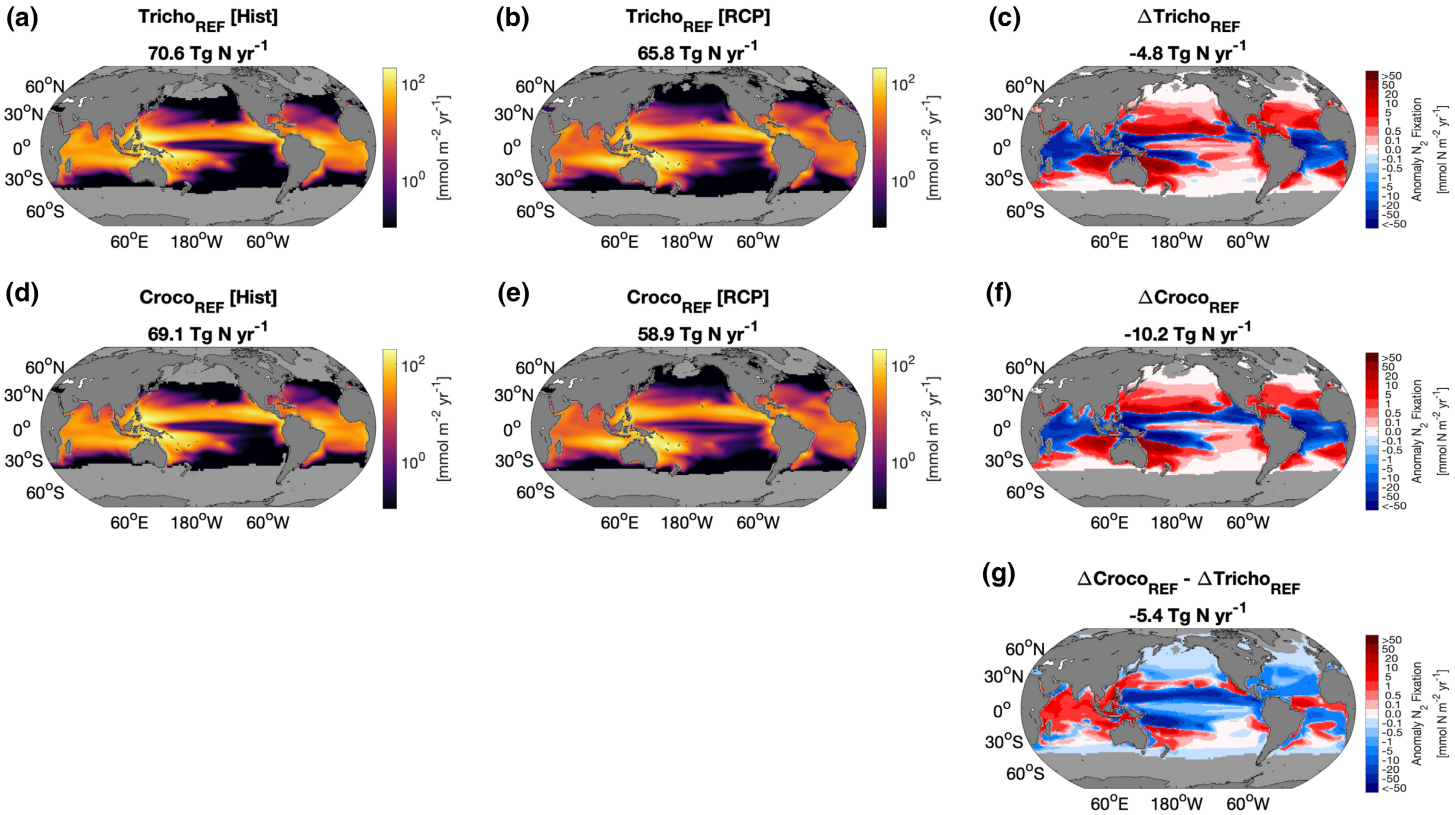
Depth integrated nitrogen fixation for *Trichodesmium* (a–c) and *Crocosphaera* (d–f) for the historical period (1996–2005; a and d) and the RCP8.5 scenario (2091–2100; b and e). Also shown are the climate change signal of nitrogen fixation (RCP—Historical; c and f) and the anomaly of the climate change signals comparing *Crocosphaera* and *Trichodesmium* (g). Values above the maps indicate globally integrated values of nitrogen fixation (a, b, d and e) and the global anomaly of nitrogen fixation (c, f, and g).

Globally, total N_2_ fixation is projected to decrease over the next century for both *Trichodesmium* and *Crocosphaera*. The decline in N_2_ fixation is stronger for *Crocosphaera* than for *Trichodesmium* with integrated N_2_ fixation decreasing from 69.1 to 58.9 Tg N year^−1^ (−15% or −10.2 Tg N year^−1^) and from 70.6 to 65.8 Tg N year^−1^ (−7% or −4.8 Tg N year^−1^), respectively (Figures 3a and 4). These declines in N_2_ fixation for the reference simulations fell within the projected trends of 9 CMIP5 models (−50.1 to +58.0 Tg N year^−1^; Wrightson & Tagliabue, 2020). In contrast, the prior diagnostic modelling predicted that N_2_ fixation would increase for both organisms by the end of the century (Jiang et al., 2018; Yang et al., 2021). The distinction with our results arises due to the prognostic representation of both top‐down (e.g., grazing and mortality) and bottom‐up (e.g., multiple limiting nutrients and competition with non‐diazotrophic phytoplankton) drivers on marine diazotrophy within a complex ESM that includes multiple competing PFTs. Regional differences in our model results were present with the Atlantic and Indian oceans responding in the same direction as the global trend for both diazotrophs (Figure 3b,d), while in the Pacific, the response was more variable. By the end of century, Pacific Ocean N_2_ fixation for *Crocosphaera* had declined by 14%, but for *Trichodesmium*, this decline was reduced and delayed relative to *Crocosphaera* with only a 3% decline occurring at the end of the century (Figure 3c). Thus, differences in global N_2_ fixation trends between each diazotroph PFT are driven by the Pacific Ocean. Our results suggest that rates of N_2_ fixation by *Trichodesmium* may be more resilient to change in the future Pacific Ocean than *Crocosphaera*. At the global scale, comparing the reference simulations to the model simulations without temperature dependent EUEs demonstrates the impact of accounting for the effect of temperature on EUEs. We find weaker declines in most of the basins for *Trichodesmium* when temperature dependent EUEs were accounted for. For *Crocosphaera*, accounting for the thermal impacts on the EUEs appears to be more beneficial in the Atlantic and Indian Oceans, whereas globally and in the Pacific Ocean, the decline in N_2_ fixation is similar or slightly stronger for Croco_REF_ simulation compared to Croco_NoEUE_ (Figure 3). Applying the lower Fe cost to N_2_ fixation for *Crocosphaera* (CrocoLowFe_REF_, Table 2) resulted in slightly higher magnitudes of global N_2_ fixation (+1.3 Tg N year^−1^) compared to Croco_REF_ simulation; however, broadly similar trends in nitrogen fixation were observed for both simulations (Figures S1 and S2). Global net primary productivity (NPP) also declined in all simulations by 5.1–6.1 Pg C year^−1^ (7.3%–9.1%). However, Tricho_REF_ resulted in a slightly lower decline of up to 0.8 Pg C year^−1^ compared to the Tricho_NoEUE_ simulation, driven primarily by changes in the Pacific Ocean and was likely responding to the enhanced N_2_ fixation in this basin. Whereas, for *Crocosphaera*, both Croco_REF_ and Croco_NoEUE_ produced similar declines in NPP. Our results suggest an overall decrease in future ocean N_2_ fixation, which contrasts with predictions of an increase in terrestrial N_2_ fixation (Davies‐Barnard et al., 2022).

### 3.2 Spatial patterns of marine nitrogen fixation

The spatial distribution of the N_2_ fixation climate signal (defined as the change in depth integrated N_2_ fixation between 1996–2005 and 2091–2100) in the reference simulation was broadly similar for both diazotrophs, with increases in the high latitudes and decreases at low latitudes (Figure 4c,f). The Pacific Ocean response is more complicated than that of the Atlantic and Indian basins, especially in the equatorial Pacific upwelling region where N_2_ fixation is also responding to projected changes to the fixed N inventory which controls the competitive niche for diazotrophs, relative to faster growing but non‐N_2_‐fixing plankton (Figure 4c,f). When the N_2_ fixation climate signal is compared between the two diazotrophs, it is apparent that *Crocosphaera* shows an amplified response, relative to *Trichodesmium* (Figure 4g). Overall, the spatial pattern of N_2_ fixation is broadly similar for the two diazotrophs, which indicates that, at least for our model, the explicit representation of only one oligotrophic diazotroph PFT may be sufficient. This is to be expected however, as apart from the different thermal performance curves for growth and EUEs, both diazotrophs have the same assumptions for minimum quotas, light limitation, and grazing pressures. Moreover, the current version of the model does not allow for competition between the two diazotrophs and, so, the role of competition for resources has not been assessed. Reducing the Fe cost of N_2_ fixation for *Crocosphaera* resulted in increased N_2_ fixation in Fe limited regions of the S. Atlantic, Pacific, and Indian oceans due to reduced Fe limitation and decreased N_2_ fixation in macronutrient limited regions of the Atlantic, N. Pacific and S. Indian oceans relative to Croco_REF_ model likely due to enhanced competition and increased macronutrient limitation (Figure 1 and Figure S2g,h). This highlighted the advantage that is gained by *Crocosphaera* in Fe limited region when the Fe cost of N_2_ fixation is reduced. The CrocoLowFe_REF_ simulation resulted in a similar spatial distribution in the N_2_ climate signal compared to Croco_REF_ (Figure S2c,e). Direct comparison of the N_2_ fixation climate signals however revealed that the reduced Fe cost of N_2_ fixation broadly resulted in weaker increases and decreases compared to the *Crocosphaera* with the higher Fe cost of N_2_ fixation (Figure S2i).

### 3.3 Identifying the drivers controlling the change in nitrogen fixation

The aim of this study was to identify how climate change, in particular, warming, can impact patterns of N_2_ fixation and which drivers were controlling the N_2_ fixation response regionally. To do this, we employed a simple environmental grouping approach based on how the model incorporates a hierarchy of controls on diazotrophy. We used the different thermal optima for growth, IUE, and PUE from the thermal performance curves. An alternative approach would be to conduct a series of exhaustive sensitivity experiments with the model to probe how the model results are affected by different assumptions. However, due to the coupled nature of fixed N supply and biogeochemical cycling, the system is not in a simple linear state, and unexpected and complex non‐linear feedbacks can emerge. Hence, the simple environmental grouping approach is more appropriate to extracting the first order controls.

**FIGURE 5:**
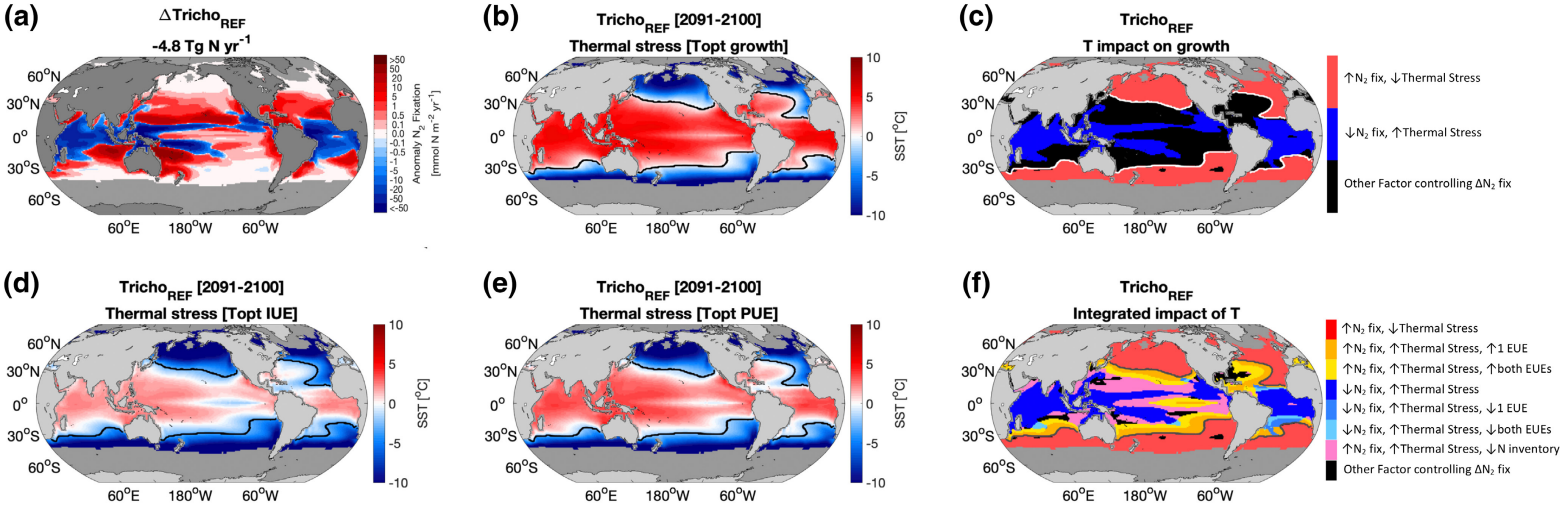
Environmental grouping based upon the effects of temperature on *Trichodesmium*: (a) climate signal of nitrogen fixation (2091–2100)—(1996–2005), (b) diazotroph thermal stress associated with growth, (c) cluster map indicating regions where the nitrogen fixation signal can be explained by the direct impacts of *T* on growth to define the niche. Thermal stress associated with (d) iron use efficiency and (e) phosphorus use efficiency. (f) Cluster map showing regions where the nitrogen fixation signal can be explained by the integrated impact of temperature on the niche and physiology of *Trichodesmium*. For the cluster map, regions explained by changing niche: Red (promoting growth/N_2_ fix), blue (restricting growth/N_2_ fix) and pink (decreasing N inventory due to stratification opening competitive niche for diazotrophy, regions where the nitrogen fixation signal can be explained by changing physiology: Shades of orange (1 or both elemental use efficiencies [EUEs] more efficient) and shades of blue (1 or both EUEs decreasing leading to nutrient limitation). Regions where other factors other than temperature are controlling the nitrogen fixation signal are coloured in black. Contours show regions where thermal optimum for growth is surpassed.

The direct impact of changes in temperature on diazotroph thermal performance through changes in diazotroph growth rates was able to explain 55%–59% of the N_2_ fixation climate signal. Globally, SST increases by between 1 to 12°C by 2091–2100 under the high emissions RCP8.5 scenario. If temperature surpasses the thermal optimum for growth, the diazotroph will experience thermal stress (red regions, Figure 5), which would decrease maximum growth and N_2_ fixation rates. If the temperature is below the thermal optimum for growth (blue regions, Figure 5b), the diazotroph would not be thermally stressed and so growth and N_2_ fixation rates would increase with warming leading to an expanded thermal niche. We evaluated the role of temperature using the monthly maximum SST during 2091–2100. Combining the spatial maps of the change in N_2_ fixation (Figure 5a) and thermal stress (quantified using the difference between SST and Topt) associated with diazotroph growth (Figure 5b), two regimes could be identified. The first regime was assigned to the regions where the diazotroph was thermally stressed (i.e., SST > Topt) and, as expected, N_2_ fixation was restricted (Blue regions, low latitudes). This regime represented 19.3% and 22.5% of the niche of *Trichodesmium* and *Crocosphaera*, respectively (Figure 5c; Table 3). The second regime was associated with regions where the diazotroph was not thermally stressed (i.e., SST < Topt) and N_2_ fixation increased as expected due to warming (red region, high latitudes = expanding thermal niche) and accounted for 35.9% and 28.7% of the niche of *Trichodesmium* and *Crocosphaera* respectively (Figure 5c; Table 3). This assessment of the effect of temperature on diazotroph thermal performance in regard to growth left almost half of the ocean (black region, 41% and 45% of the niche of *Trichodesmium* and *Crocosphaera* respectively) in which the change could not be explained (Figure 5c). In these regions, despite being thermally stressed (SST > Topt), N_2_ fixation increased. This was due to temperature driven changes in diazotroph physiology mediated through altered EUEs in response to warming, explaining the climate trend in N_2_ fixation for around a quarter of the diazotroph's niche, with the remainder being attributed to the emergence of a new N‐limited niche, which promoted diazotrophy. As discussed above, temperature can also affect rates of N_2_ fixation by altering the efficiency of enzymes, and the EUEs can be used to explore this. If temperature surpasses the thermal optimum of the EUEs (i.e., SST > Topt EUE), the declining EUEs (e.g., due to enzymes denaturing) lead to increased nutrient demand and enhanced nutrient limitation (red areas, Figure 5d,e). Alternatively, if the temperature remains below the thermal optimum of the EUEs (i.e., SST < Topt EUE), then EUEs increase with ocean warming, alleviating nutrient limitation, and promoting both growth and N_2_ fixation despite reduced maximum growth rates (blue areas, Figure 5d,e).

**TABLE 3:**
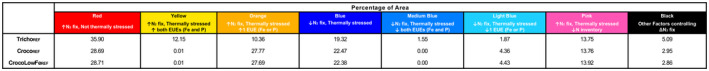
Percentage of diazotroph niche that can be explained by each environmental group

This concept of temperature adjusted EUEs can be used to further explain the N_2_ fixation trend in regions not explained by the temperature effects on the thermal niche of diazotrophy (black region, Figure 5c). First, a regime can be identified where one or both EUEs for each diazotroph have increased due to warming, and N_2_ fixation rates increased due to reduced nutrient limitation despite diazotroph growth being thermally stressed (shades of orange/yellow), this regime accounted for 22.5% and 27.8% of the niche of *Trichodesmium* and *Crocosphaera*, respectively (Figure 5f; Table 3). A second regime displayed reduced EUEs in response to warming alongside declining N_2_ fixation, and despite no thermal stress on diazotroph growth, N_2_ fixation declined likely due to enhanced nutrient demand (shade of blue areas): this regime represented 3.4% and 4.4% of the niche of *Trichodesmium* and *Crocosphaera* respectively (Figure 5e; Table 3). For *Trichodesmium*, both Fe and P use efficiencies increase, but for *Crocosphaera* only P use efficiency increases within the black region (Figure 5d,e). Thus, changing EUEs due to warming explain the response of N_2_ fixation in around a quarter of their niche. Finally, temperature changes due to climate can also indirectly impact diazotrophy through the decline in the upper 100 m N inventory due to enhanced vertical stratification creating a niche for diazotroph in regions with excess P relative to N. The decrease in the N inventory leads to increased N limitation of fast growing non‐diazotroph PFTs, providing the slower growing diazotrophs with a competitive advantage. This new niche for diazotrophy emerged largely in the Pacific Ocean (pink area, Figure 5f). This new competitive niche explained 13.8% of the niche for both diazotrophs (Figure 5f; Table 3). Reducing the Fe cost of N_2_ fixation for *Crocosphaera* produced very similar results to those of the standard *Crocosphaera* model (Table 3).

Overall, by applying this environmental grouping approach, 95% and 97% of the spatial N_2_ fixation signal can be attributed to drivers for *Trichodesmium* and *Crocosphaera*, respectively. Around half of the signal is attributed to the effect of temperature on diazotroph growth defining a thermal niche for diazotrophy, a quarter due to the effect of warming via changing EUEs and the remainder due to competition with non‐N_2_‐fixing plankton in N limited regions. The small fraction of the ocean (at most 5%) that cannot be attributed to these factors are being controlled by other factors such as grazing, light availability or community shifts (Table 3).

**FIGURE 6:**
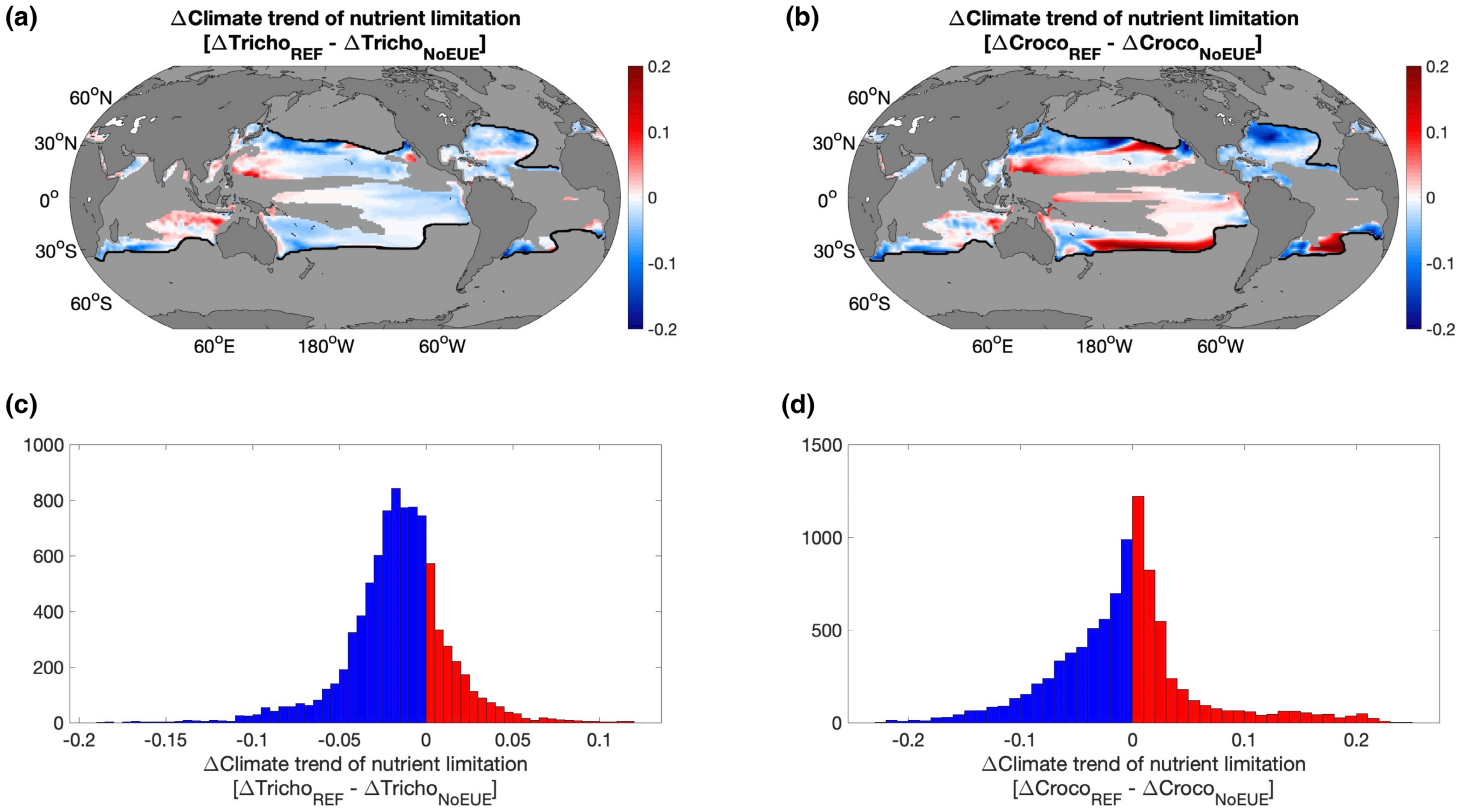
Anomaly of the climate trend of nutrient limitation for Tricho_REF_ (a and c) and Croco_REF_ (b and d) compared to the model runs without any elemental use efficiency parameterisation (Tricho_NoEUE_ and Croco_NoEUE_). Maps and histograms focus on the black environmental group where the nitrogen fixation anomaly could not be explained by the temperature effects on the diazotrophs thermal niche. Blue indicates decreasing nutrient limitation and red indicates increasing nutrient limitation.

In our model, we can further examine how the changing diazotroph physiology due to the effect of temperature on EUEs was reflected in their overall nutrient limitation. Here, we focus on regions where the climate trend in N_2_ fixation rates was not simply due to the temperature effect on the thermal niche of diazotrophy (i.e., the black region in Figure 5c). We isolated this area and compared the climate trend of diazotroph nutrient limitation for both *Trichodesmium* and *Crocosphaera* to the model runs where no temperature‐EUE parameterisation was present (Figure 6a,b). In general, for the majority of this region, nutrient limitation was decreasing in the reference simulations as expected from our grouping approach and consistent with warming effects on EUEs (Figure 6c,d). An interesting point to note is that for *Trichodesmium*, nutrient limitation decreased broadly across the whole of the black region, whereas for *Crocosphaera*, nutrient limitation decreased in the Atlantic but not in the Pacific (Figure 6b). This likely arises because only the PUE increases for *Crocosphaera* in this region, providing an advantage in the P limited Atlantic, but no advantage in the Fe limited Pacific, leading to increased nutrient limitation (Figures 1b and 6b). For *Trichodesmium* however, both Fe and P EUEs increase and broad decreases in nutrient limitation are observed across both basins (Figure 6a).

**FIGURE 7:**
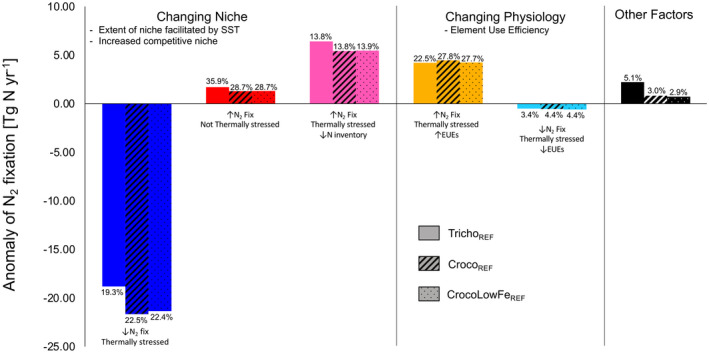
Contribution of each environmental group to the climate signal of nitrogen fixation for Tricho_REF_ (solid), Croco_REF_ (hatched) and CrocoLowFe_REF_ (dotted). Temperature impacts on the niche of diazotrophy: Blue (restricting growth/N_2_ fix), red (promoting growth/N_2_ fix), and pink (decreasing N inventory due to stratification opening a competitive niche for diazotrophy). Temperature impacts on physiology: Orange (1 or both elemental use efficiencies [EUEs] more efficient) and cyan (1 or both EUEs decreasing leading to nutrient limitation). Black bars indicate the environmental group which cannot be explained by temperature. Percentages show how much of the area of the diazotroph's geographic niche each group occupies.

Ultimately, the changes in N_2_ fixation in our model experiments are associated with either a change to the geographic niche of diazotrophy or by changes to their physiology regarding EUEs and nutrient limitation via alterations to Fe and/or P EUEs. A changing niche explains the impact of climate on diazotrophy over the majority of the ocean area, either due to thermal stress leading to a shrinking niche, thermal expansion of the niche to higher latitudes or a competitive advantage for diazotrophs in newly N‐limited regions. In addition, the regions associated with changing diazotroph physiology, either due to increasing or decreasing EUEs, explain N_2_ fixation trends for around a quarter of the diazotroph niche. If assessed in terms of the contribution of each regime to the overall integrated change in N_2_ fixation (Figure 7), we see the dominant effect of the decline of ~20 Tg N year^−1^ due to thermal stress. Around half of this is compensated for by both a new niche in newly N‐limited regions and improved EUEs under warming (~5 Tg N year^−1^ each). In absolute terms, the expanding thermal niche to higher latitudes only plays a minor role in our model experiments as the temperatures in these regions remain suboptimal for both diazotrophs. This indicates that both the integrated effects of temperature on the niche and physiology of marine diazotrophs need to be accounted for in ESM to fully assess the impact of warming on the total rates of marine N_2_ fixation that drive the broader biogeochemical consequences.

### 3.4 Responses to warming

**FIGURE 8:**
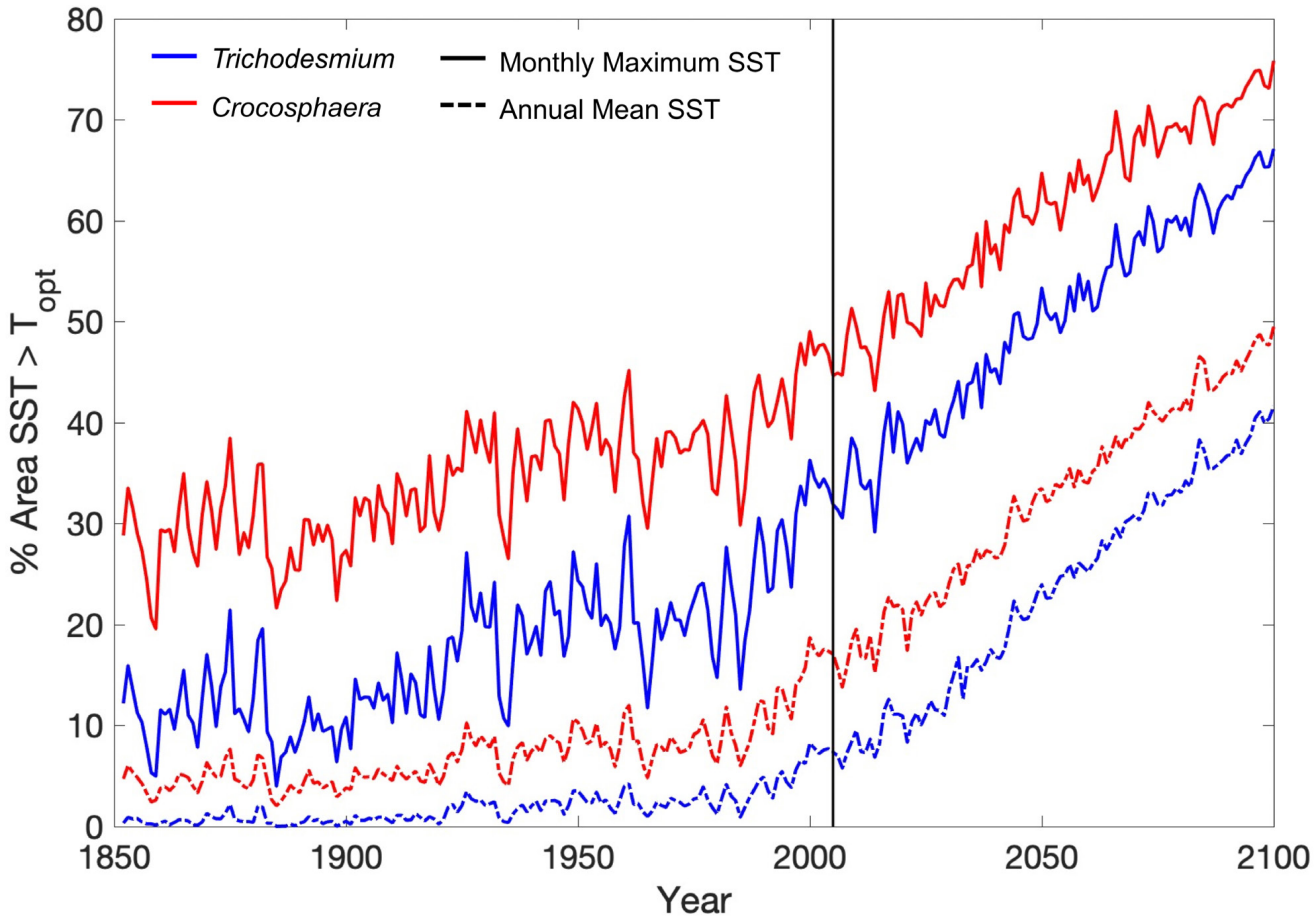
Percentage of the area of the diazotroph's niche where temperature has surpassed the thermal optimum for growth for both *Trichodesmium* (blue) and *Crocosphaera* (red) using the monthly maximum sea surface temperature (SST) (solid lines) and the annual mean SST (dashed lines). Black vertical line indicates the end of the historical period and the start of the RCP8.5 forcing.

The current version of the model assumes that the thermal performance of the modelled diazotroph is fixed and neglects any thermal evolution. This means that once their maximum thermal threshold is surpassed by rising SST, they are excluded, which drives a large decline in both their niche and absolute N_2_ fixation rates. However, biology is highly dynamic, with both evolution and adaptation likely to occur. A recent experimental evolution study comparing *Trichodesmium* with *Crocosphaera* under sustained thermal selection suggested that the former showed little capacity to adapt to warming, but instead relied on non‐genetic plasticity to meet temperature challenges (Qu et al., 2022). *Crocosphaera* however exhibited a limited ability to adapt to supraoptimal warming supported by a suite of specific genetic changes, suggesting that evolutionary capacity may need to be considered at least for this diazotroph (Qu et al., 2022). This may imply that in the future, *Crocosphaera* may more readily adapt to warming compared to *Trichodesmium*, enabling *Crocosphaera* to potentially occupy the niche that *Trichodesmium* has been thermally excluded from. However, more experimental work is required to better understand how both diazotroph groups adapt to warming before this evolutionary capacity can be incorporated into ESMs. Our results provide a gauge as to the rate at which temperature will exceed the thermal optimum of growth for *Trichodesmium* and *Crocosphaera* and how this compares to experimental studies of thermal adaptation of both diazotrophs. Our results imply that, based on the monthly maximum SST, the thermal optimum for growth has already been surpassed for much of the low latitude ocean by the end of the historical period for both diazotrophs (Figure 8). At the end of the historical period (1996–2005) the area of the diazotroph's niche where they are experiencing thermal stress (i.e. where temperature exceeds the thermal optimum) was 6%–31% for *Trichodesmium* and 15%–43% for *Crocosphaera* (Figure 8; Table 4).

**TABLE 4:**
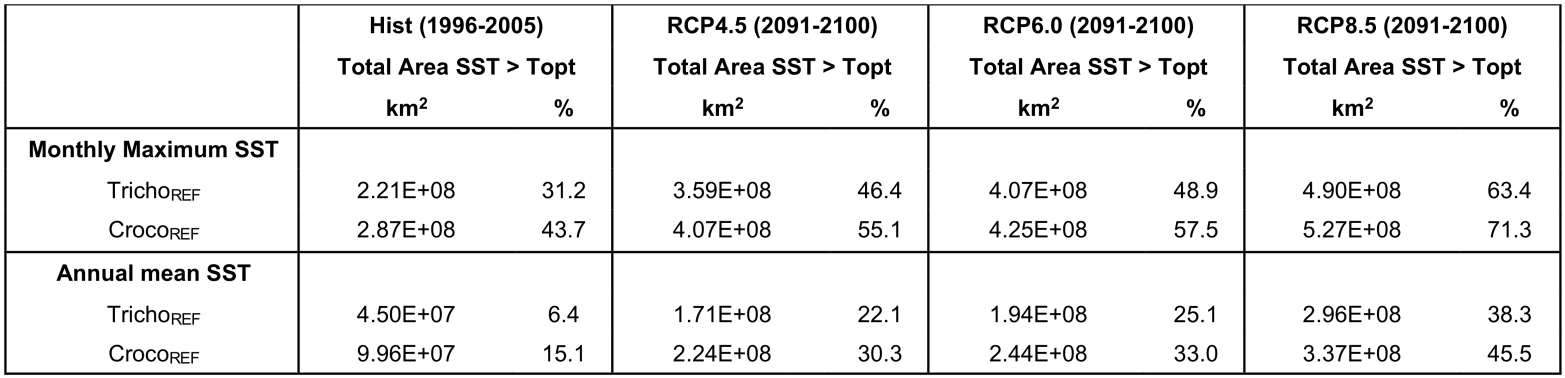
Area of the thermal niche of diazotrophy where thermal stress is occurring for *Trichodesmium* and *Crocosphaera* for both the monthly maximum temperature and annual mean temperature for the historical period (1996–2005) and for several RCP climate forcing scenarios (RCP4.5, RCP6.0 and RCP8.5 (2091–2100))

By the end of the century, the area of thermal stress roughly doubles for both diazotrophs, and for most of the low latitudes, the diazotrophs are thermally stressed within 10 years of the high emissions RCP8.5 scenario (Figure 8; Table 4). This indicates that if diazotrophs cannot adapt to warming in the future they may be excluded from broad regions of the low latitudes. If the annual mean temperature is used the area of thermal stress is ~25% less than if the monthly maximum temperature is used but the outlook is the same (Table 4). The thermal niche of each diazotroph is determined by the specific thermal performance curves for growth that define the thermal thresholds for diazotroph growth. Due to the colder temperatures at high latitudes in the historical period (1986–2005), *Trichodesmium* and *Crocosphaera* were excluded from 31% to 40% and 36% to 49% of the ocean, respectively. With future warming, this area decreases over the coming century by up to 8% for *Trichodesmium* or by up to 10% for *Crocosphaera*, as diazotrophs expand their niche into higher latitudes (Table S1). In this study, the monthly maximum temperature was used as these are the actual conditions the diazotrophs will experience in the model. Our estimates of diazotroph thermal stress are based on a strict temperature criterion that states if the specific thermal optimum of diazotroph growth is surpassed then the diazotroph is thermally stressed. However, a recent modelling study investigating optimal growth of *Trichodesmium* defined optimal growth conditions as those that allow growth rates of >0.25 day^−1^ and suggested that when considering the combined impact of temperature, light, and Fe availability, the area of optimal conditions experienced by *Trichodesmium* may increase by up to 173% by 2100 (Boatman et al., 2020). The study by Boatman et al. (2020) also indicated that the thermal niche of *Trichodesmium* will likely expand at high latitudes and reduce in equatorial regions, agreeing with the findings of this study. It is worth noting however, that under lower emissions scenarios, the associated reduction in warming would reduce the extent of thermal stress for both diazotrophs by 12%–17% compared to the high emissions RCP8.5 scenario (Table 2; Figure S3). Any reduction in warming and thermal stress under alternative emissions scenarios would lead to a lesser degree of thermal exclusion and enable diazotrophs to remain at low latitudes, promoting N_2_ fixation in these regions (Table 4). At high latitudes however, any reduction in warming under lower emissions trajectories would restrict the thermal expansion and greater N_2_ fixation seen under the high emissions scenario.

## 4 WIDER IMPLICATIONS AND FURTHER WORK

Currently, the model can only represent one diazotroph (either *Trichodesmium* or *Crocosphaera*) at a time, which does not allow for competition between the two diazotrophs to occur. It would therefore be interesting to implement both diazotrophs into the model as co‐existing PFTs to investigate how competition between the two organisms in the model impacts rates of N_2_ fixation. In the ocean, *Trichodesmium* and *Crocosphaera* would compete for resources, particularly Fe and P. Both microbes are adapted to low P environments and are able to access dissolved organic phosphate (DOP) alleviating P limitation (Dyhrman et al., 2002; Dyhrman & Haley, 2006). Unlike *Crocosphaera*, *Trichodesmium* is also able to deploy high affinity P strategies, enabling growth on polyphosphate and phosphonates providing a competitive advantage and potentially reducing competition (Dyhrman et al., 2006; Orchard et al., 2010). Both organisms also occur at different depths with *Crocosphaera* generally present deeper in the water column to avoid photoinhibition, while *Trichodesmium* is better able to cope with high irradiance levels and prefers the high light surface waters, and so spatial separation may also prevent competition (Andresen et al., 2010; Inomura et al., 2019).

As with all global ocean biogeochemical models, nutrient limitation in our model is determined by the most limiting nutrient (either Fe or P for diazotrophs). However, throughout regions of the Atlantic and Pacific Oceans, diazotrophs have been observed to be exposed to simultaneous Fe and P co‐limitation (Cerdan‐Garcia et al., 2021; Mills et al., 2004; Wen et al., 2022), with a recent metaproteomic study suggesting that Fe–P co‐stress may be considered the normal conditions that *Trichodesmium* is exposed to in the North Atlantic (Held et al., 2020). Under laboratory conditions, enhanced growth and N_2_ fixation rates were observed for both *Trichodesmium* and *Crocosphaera* when each diazotroph was exposed to Fe and P co‐limitation (Garcia et al., 2015; Walworth et al., 2016). These results suggest that both diazotrophs have adapted for growth in Fe and P co‐limited conditions, highlighting the need to incorporate nutrient co‐limitation in future model studies.

Although our model is unusual in representing both *Trichodesmium* and *Crocosphaera* responses to climate change, they are not the only diazotrophs in the ocean, and molecular techniques have identified a wide diversity of diazotrophic organisms co‐existing in the ocean including both autotrophic and heterotrophic diazotrophs (Zehr & Capone, 2020). One of particular interest is the symbiotic unicellular cyanobacteria, UCYN‐A which may be more prevalent globally than either *Trichodesmium* or *Crocosphaera* (Martinez‐Perez et al., 2016). Generally, UCYN‐A occupies higher latitudes and coastal regions, including areas with substantial standing stocks of nitrate, which may allow the niche of diazotrophy to expand past the tropics and sub‐tropics within the model (Zehr & Capone, 2020). UCYN‐A would be an important candidate to include in the model, but to be able to implement UCYN‐A into the model, observational/laboratory data on growth and EUE would be required which is currently challenging, as cultures are not yet widely available.

We have focused upon the combined effects of warming on marine N_2_ fixation but other potentially important drivers of change are grazing by zooplankton and ocean acidification. Zooplankton have been observed to consume diazotrophs in the ocean (Horii et al., 2018; Turk‐Kubo et al., 2018), and it has been suggested by a recent modelling study that grazing has the potential to control patterns of marine N_2_ fixation (Wang et al., 2019). The potential control of grazing upon diazotrophs is likely to differ between *Trichodesmium* and *Crocosphaera*. *Trichodesmium* has been observed to produce toxins which may reduce grazing pressures by limiting the number of predators that can consume *Trichodesmium* (LaRoche & Breitbarth, 2005). On the other hand, microzooplankton have been observed to graze upon smaller unicellular diazotrophs, which may imply that *Crocosphaera* may be more susceptible to grazing (Turk‐Kubo et al., 2018). The difference in grazing pressure experienced by each diazotroph could be used as a differential control within the model. The current grazing parameterization within the model causes grazing on diazotrophs to be highly positively correlated with diazotroph biomass. By altering the zooplankton grazing preference for diazotrophs within the model, the grazing pressure experienced by the diazotrophs will also change, with decreased grazing preference promoting N_2_ fixation and increased grazing preference restricting N_2_ fixation rates. Ocean acidification also has the potential to shape patterns of diazotrophy in the future. Currently, the model does not account for the impact of increasing CO_2_ on marine diazotrophs. This result of ocean acidification can promote diazotrophy under Fe replete conditions (Fu et al., 2008; Walworth et al., 2016) and may play a role in regions like the North Atlantic that are Fe replete. Further work is needed to integrate the range of drivers that operate alongside warming to shape the response of diazotrophs to climate change. It would therefore be interesting to investigate how both ocean acidification and the role of top‐down controls such as grazing pressure interact with warming driven impacts to shape the patterns of N_2_ fixation in the future.

Finally, the model used in this study is only accounting for the impact of temperature on diazotroph physiology in regard to N_2_ fixation. As has been shown in this study, two diazotrophs with different thermal performance curves respond differently to warming; however, other processes such as photosynthesis and respiration involve enzymes that may respond differently to warming compared to those associated with N_2_ fixation. In the studies that measured the EUEs of N_2_ fixation, the carbon fixation EUEs were also measured and show slight differences compared with the N_2_ fixation EUEs, which may lead to regional shifts in the diazotrophy niche for both *Trichodesmium* and *Crocosphaera* (Jiang et al., 2018; Yang et al., 2021). A similar approach using EUEs could be applied to other PFTs within the model. The thermal windows of non‐diazotroph phytoplankton have been found to range from temperatures as cold as <5°C to warmer temperatures of 35°C and each species has a specific thermal optimum for growth (Boyd et al., 2013). This implies that the thermal performance of phytoplankton will be highly variable throughout the ocean with each phytoplankton experiencing different levels of thermal stress based upon their adaptation to temperature. Warming will therefore impact upon growth rate and EUEs of different phytoplankton, shaping patterns of nutrient limitation and ultimately defining their environmental niche. The response of different PFT to warming will alter patterns of resource availability and competition influencing ocean biogeochemistry. Therefore, to gain a more complete understanding of how warming will impact ocean biogeochemistry it is essential to include temperature adjusted EUEs for both other PFTs and for other processes such as carbon fixation.

## 5 CONCLUSIONS

In this study, we have developed a new state‐of‐the‐art explicit diazotroph model for PISCES QUOTA to investigate how diazotroph thermal fitness shapes patterns of marine N_2_ fixation. The model can switch between two prevalent marine diazotrophs, *Trichodesmium* and *Crocosphaera*, and uses observed thermal performance curves of growth and N_2_ fixation EUEs to represent the thermal fitness of both diazotrophs. This enables the integrated effects of warming on both the niche and physiology of both diazotrophs to be assessed and identify how this shapes the response marine N_2_ fixation to climate change. We have shown that both diazotroph‐specific thermal performance curves and EUEs impact the response of N_2_ fixation to climate change. N_2_ fixation is predicted to decrease globally for both diazotrophs, but regional differences occur particularly in the Pacific Ocean, which acts to shape the global response of *Trichodesmium* and *Crocosphaera* to climate change and the knock‐on effects for NPP. Both diazotrophs exhibit broadly similar spatial patterns of N_2_ fixation with increases in the high latitudes driven by thermal expansion and decreases in the low latitudes due to thermal exclusion. The integrated impact of temperature on marine diazotrophy explained 95%–97% of the N_2_ fixation climate change signal, with two groups of drivers emerging, those associated with a change in the diazotroph's niche and those associated with a change in diazotroph physiology. Decreases in N_2_ fixation were dominated by a change in the diazotroph niche, while increases were driven by a combination of both a changing niche and changing physiology. With temperatures rising diazotroph thermal stress will increase, and it is predicted that by the end of the century, the area of thermal stress will double. This implies that if diazotrophs cannot adapt rapidly enough to increasing temperatures they may be excluded from large regions of the low latitude ocean. Overall, we have performed a holistic consideration of the impact of warming on diazotrophy, highlighting that the effects of temperature on diazotroph thermal fitness will interact to shape the response of N_2_ fixation to climate change, which will have important implications for marine primary productivity in the future.

## AUTHOR CONTRIBUTIONS

Lewis Wrightson, Nina Yang, David A. Hutchins, and Alessandro Tagliabue designed the study. Lewis Wrightson and Alessandro Tagliabue developed and coded the model. Nina Yang and David A. Hutchins provided observations of diazotroph growth rates and EUEs. Lewis Wrightson and Alessandro Tagliabue analyzed and interpreted data. All authors provided discussion on the model parameterization. Lewis Wrightson conducted the model simulations and performed the data analysis. All authors contributed to the manuscript and approved the submitted version.

## CONFLICT OF INTEREST

The authors have declared no conflict of interest.

## Supporting information


Appendix S1

## Data Availability

Model output is available on Zenodo at https://doi.org/10.5281/zenodo.6541954. The full model description is included in the supplementary material. The model code and initialization file are available on GitHub at https://github.com/lewiswrightson/PISCES‐QUOTA‐P6Z.
