# Supplementary material for: Integrating the impact of global change on the niche and physiology of marine nitrogen‐fixing cyanobacteria
Source: Glob Chang Biol. 2022 Sep 13;28(23):7078–93. doi: 10.1111/gcb.16399 (PMC9826025; doi:10.1111/gcb.16399)
Supplement: Supplementary file 1 — Appendix S1 [file GCB-28-7078-s001.docx]

**Supplementary Material**

1. **Model Description**

For this study we developed a new explicit diazotroph phytoplankton functional type (PFT) for the optimal allocation-based PISCES QUOTA ESM (Kwiatkowski et al., 2018). The PISCES QUOTA model includes 3 PFT and 2 zooplankton grazers, phytoplankton stoichiometry is allowed to vary, and phytoplankton growth is limited by temperature, light and nutrients. Here, we describe the new explicit diazotroph model based upon observed diazotroph thermal performance curve and nutrient requirements. The model allows the diazotroph stoichiometry to vary, accounts for optimal allocation and the competition for resources between diazotrophs and the other phytoplankton types within the model, as well as zooplankton grazing of diazotroph biomass. The model is able to switch between two prevalent marine diazotrophs, *Crocosphaera* and *Trichodesmium*, and is able to account for the indirect effects of warming on elemental use efficiency.

- 1. **Diazotroph Carbon Biomass**

Production

Biosynthesis

Mortality

Aggregation

$\frac{\boldsymbol{\partial}\boldsymbol{P}_{\boldsymbol{C}}^{\boldsymbol{dz}}}{\boldsymbol{\partial t}}$ **=** $\left( \boldsymbol{1-}\boldsymbol{\delta}^{\boldsymbol{dz}} \right)\boldsymbol{\mu}_{\boldsymbol{P}}^{\boldsymbol{dz}}\boldsymbol{P}_{\boldsymbol{C}}^{\boldsymbol{dz}}\boldsymbol{-}\boldsymbol{\zeta}_{\boldsymbol{NO}\boldsymbol{3}}^{\boldsymbol{dz}}\boldsymbol{V}_{\boldsymbol{NO}\boldsymbol{3}}^{\boldsymbol{dz}}\boldsymbol{-}\boldsymbol{\zeta}_{\boldsymbol{NH}\boldsymbol{4}}^{\boldsymbol{dz}}\boldsymbol{V}_{\boldsymbol{NH}\boldsymbol{4}}^{\boldsymbol{dz}}\boldsymbol{-}\boldsymbol{\zeta}_{\boldsymbol{NFIX}}^{\boldsymbol{dz}}\boldsymbol{V}_{\boldsymbol{NFIX}}^{\boldsymbol{dz}}\boldsymbol{-}\boldsymbol{m}^{\boldsymbol{dz}}\frac{\boldsymbol{P}_{\boldsymbol{C}}^{\boldsymbol{dz}}}{\boldsymbol{K}_{\boldsymbol{m}}\boldsymbol{+}\boldsymbol{P}_{\boldsymbol{C}}^{\boldsymbol{dz}}}\boldsymbol{P}_{\boldsymbol{C}}^{\boldsymbol{dz}}\boldsymbol{-sh}\boldsymbol{\times}\boldsymbol{w}^{\boldsymbol{dz}}\mathbf{(}\boldsymbol{P}_{\boldsymbol{C}}^{\boldsymbol{dz}}\boldsymbol{)}^{\boldsymbol{2}}$ $\boldsymbol{-}\boldsymbol{g}^{\boldsymbol{Z}}\left( \boldsymbol{P}_{\boldsymbol{C}}^{\boldsymbol{dz}} \right)\boldsymbol{Z-}\boldsymbol{g}^{\boldsymbol{M}}\left( \boldsymbol{P}_{\boldsymbol{C}}^{\boldsymbol{dz}} \right)\boldsymbol{M}$

(Equation S1)

Grazing

Diazotroph carbon (C) biomass ($P_{C}^{dz}$) in equation S1 is calculated from a source term of diazotroph production and several sink terms (biosynthesis, mortality, aggregation and grazing) and follows the parameterisation currently used for other phytoplankton functional types (PFT).

Production of Diazotroph Biomass:

Diazotroph production is calculated in the same way as is done for other PFTs where growth rate is multiplied by an exudation term and the diazotroph C biomass at the previous time step.

$\boldsymbol{P}_{\boldsymbol{C}}^{\boldsymbol{dz}}$ = Diazotroph C biomass (mol C L^-1^)

$\boldsymbol{\mu}_{\boldsymbol{P}}^{\boldsymbol{dz}}$ = Growth rate (d^-1^)

$\boldsymbol{\delta}^{\boldsymbol{dz}}$ = 0.05 exudation of DOC (unitless)

Biosynthesis:

Diazotroph biosynthesis accounts for the uptake of nitrate (NO_3_), ammonium (NH_4_) and dinitrogen (N_2_) as well as the associated costs of biosynthesis of each form of nitrogen (N).

$\boldsymbol{V}_{\boldsymbol{NO}\boldsymbol{3}}^{\boldsymbol{dz}}$ = Uptake of NO_3_ (mmol N L^-1^ s^-1^)

$\boldsymbol{V}_{\boldsymbol{NH}\boldsymbol{4}}^{\boldsymbol{dz}}$ = Uptake of NH_4_ (mmol N L^-1^ s^-1^)

$\boldsymbol{V}_{\boldsymbol{NFIX}}^{\boldsymbol{dz}}$ = Uptake of N_2_ through nitrogen fixation (mmol N L^-1^ s^-1^)

$\boldsymbol{\zeta}_{\boldsymbol{NO}\boldsymbol{3}}^{\boldsymbol{dz}}$ = 2.3 mol C mol^-1^ N cost of biosynthesis based on NO_3_

$\boldsymbol{\zeta}_{\boldsymbol{NH}\boldsymbol{4}}^{\boldsymbol{dz}}$ = 1.8 mol C mol^-1^ N cost of biosynthesis based on NH_4_

$\boldsymbol{\zeta}_{\boldsymbol{NFIX}}^{\boldsymbol{dz}}$ = 6.6 mol C mol^-1^ N cost of biosynthesis based on N_2_ (Pahlow et al., 2013)

$\zeta_{NFIX}^{dz}=\frac{cost of NFix (Pahlow)}{cost of N assimilation (Pahlow)}\times\zeta_{NO3}^{dz}=\frac{2}{0.7}\times2.3$ (Equation S2)

The cost of biosynthesis based on N_2_ follows Pahlow et al. (2013), it calculates the ratio of the respiration cost required for nitrogen fixation (2 mol C mol^-1^ N) and the respiration cost required for N assimilation (0.7 mol C mol^-1^ N), this is then multiplied by the cost of biosynthesis based on NO_3_ to calculate the overall cost of nitrogen fixation ($\zeta_{NFIX}^{dz}$).

Mortality and Aggregation:

Mortality and aggregation for the diazotroph are also calculated following the same approach as used for other PFTs.

**K_m_**  = 0.2 µmol C L^-1^ Half saturation constant of mortality

**m^dz^** = 0.01 d^-1^ mol^-1^ C Linear mortality rate of diazotrophs

**w^dz^** = 0.01 d^-1^ mol^-1^ C Quadratic mortality rate of diazotrophs

**sh**  = Shear rate

Grazing:

The diazotroph PFT can be grazed by both meso- and microzooplankton. For the diazotroph however, the grazing preference for meso and micro zooplankton has been reduced to 0.2 (nanophytoplankton = 0.3, diatoms = 1) and 0.4 (nanophytoplankton = 1, diatoms = 0.8 and picophytoplankton = 1) respectively to account for potential toxicity of diazotrophs to grazers (LaRoche and Breitbarth, 2005).

**g^Z^** = grazing rate of mesozooplankton (d^-1^)

**Z**  = mesozooplankton biomass (mol C L^-1^)

**g^M^**  = grazing rate of microzooplankton (d^-1^)

**M** = microzooplankton biomass (mol C L^-1^)

Diazotroph Growth Rate ($\mu_{P}^{dz})$:

Diazotroph growth rate in equation S3 is calculated using the same parameterisation as other PFTs, however the growth curve ($\mu_{max}^{dz}$) is accounted for differently.

$\boldsymbol{\mu}_{\boldsymbol{P}}^{\boldsymbol{dz}}\boldsymbol{=}\boldsymbol{\mu}_{\boldsymbol{max}}^{\boldsymbol{dz}}\boldsymbol{h}\left( \boldsymbol{L}_{\boldsymbol{day}} \right)\boldsymbol{g}^{\boldsymbol{i}}\left( \boldsymbol{Z}_{\boldsymbol{mxl}} \right)\boldsymbol{L}_{\boldsymbol{lim}}^{\boldsymbol{dz}}\left( \boldsymbol{1-}\exp\left( \frac{\boldsymbol{-}\boldsymbol{\alpha}^{\boldsymbol{dz}}\boldsymbol{\theta}^{\boldsymbol{Chl,dz}}\boldsymbol{PAR}^{\boldsymbol{dz}}}{\boldsymbol{\mu}_{\boldsymbol{max}}^{\boldsymbol{dz}}\boldsymbol{h}\left( \boldsymbol{L}_{\boldsymbol{day}} \right)\boldsymbol{L}_{\boldsymbol{lim}}^{\boldsymbol{dz}}} \right) \right)$ (Equation S3)

**h(L_day_)** = dependency of growth rate to day length (unitless)

**Z_mxl_** = mixed layer depth (m)

**g^i^** = imposes a restriction to growth rate when mixed layer depth is deeper than euphotic zone (unitless)

$\boldsymbol{L}_{\boldsymbol{lim}}^{\boldsymbol{dz}}$ = Diazotroph nutrient limitation (unitless scalar ranging from 0 to 1)

$\boldsymbol{\theta}^{\boldsymbol{Chl,dz}}$ = diazotroph Chl:C ratio (g Chl g^-1^ C)

$\boldsymbol{PAR}^{\boldsymbol{dz}}$ = Photosynthetically available radiation (W m^-2^)

$\boldsymbol{\alpha}^{\boldsymbol{dz}}$ = 2 Wm^-2^ d^-1^ (nanophytoplankton = 4 Wm^-2^ d^-1^) Initial slope of P-I curve

Allocation of Nutrients within the Cell

Within the model nutrients are allocated between surface sites ($\boldsymbol{F}_{\boldsymbol{A, S}}^{\boldsymbol{dz}}$) and internal enzymes ($1-\boldsymbol{F}_{\boldsymbol{A, S}}^{\boldsymbol{dz}}$), with *S* denoting the nutrient in question. For nutrient uptake surface sites are required and the allocation to these surface sites is calculated as follows.

$\boldsymbol{F}_{\boldsymbol{A, S}}^{\boldsymbol{dz}}\boldsymbol{=}\frac{\boldsymbol{1}}{\boldsymbol{1+}\sqrt{\frac{\boldsymbol{S}}{\boldsymbol{K}_{\boldsymbol{S}}^{\boldsymbol{dz}}}}}$ (Equation S4)

$\boldsymbol{K}_{\boldsymbol{S}}^{\boldsymbol{dz}}$ = half saturation constant for nutrient S (μmol L^-1^ for N and P, nmol L^-1^ for Fe)

- 1. **Diazotroph Nitrogen Biomass**

In this version, the diazotroph is facultative allowing both the supply of N via nitrogen fixation and the uptake of other forms of fixed N (e.g. NO_3_ and NH_4_) if they are available. Diazotroph N biomass is calculated in the same way as is done for other PFTs with an additional term for N uptake via nitrogen fixation $\boldsymbol{(V}_{\boldsymbol{NFIX}}^{\boldsymbol{dz}}\boldsymbol{)}$.

$$\frac{\boldsymbol{\partial P}_{\boldsymbol{N}}^{\boldsymbol{dz}}}{\boldsymbol{\partial t}}\boldsymbol{=}\left( \boldsymbol{1-}\boldsymbol{\partial}^{\boldsymbol{dz}} \right)\left( \boldsymbol{V}_{\boldsymbol{NO}\boldsymbol{3}}^{\boldsymbol{dz}}\boldsymbol{+}\boldsymbol{V}_{\boldsymbol{NH}\boldsymbol{4}}^{\boldsymbol{dz}}\boldsymbol{+}\boldsymbol{V}_{\boldsymbol{NFIX}}^{\boldsymbol{dz}} \right)\boldsymbol{P}_{\boldsymbol{C}}^{\boldsymbol{dz}}\boldsymbol{-}\boldsymbol{m}^{\boldsymbol{dz}}\frac{\boldsymbol{P}_{\boldsymbol{C}}^{\boldsymbol{dz}}}{\boldsymbol{k}_{\boldsymbol{m}}\boldsymbol{+}\boldsymbol{P}_{\boldsymbol{C}}^{\boldsymbol{dz}}}\boldsymbol{P}_{\boldsymbol{N}}^{\boldsymbol{dz}}\boldsymbol{-sh}\boldsymbol{\times}\boldsymbol{w}^{\boldsymbol{dz}}\boldsymbol{P}_{\boldsymbol{C}}^{\boldsymbol{dz}}\boldsymbol{P}_{\boldsymbol{N}}^{\boldsymbol{dz}}$$

$\boldsymbol{-}\boldsymbol{g}^{\boldsymbol{Z}}\left( \boldsymbol{P}^{\boldsymbol{dz}} \right)\boldsymbol{Z}\boldsymbol{Q}_{\boldsymbol{N}}^{\boldsymbol{dz}}\boldsymbol{-}\boldsymbol{g}^{\boldsymbol{M}}\left( \boldsymbol{P}^{\boldsymbol{dz}} \right)\boldsymbol{M}\boldsymbol{Q}_{\boldsymbol{N}}^{\boldsymbol{dz}}$ (Equation S5)

Uptake of N:

$\boldsymbol{V}_{\boldsymbol{NH}\boldsymbol{4}}^{\boldsymbol{dz}}\boldsymbol{=}\boldsymbol{V}_{\boldsymbol{N,max}}^{\boldsymbol{dz}} \boldsymbol{x}_{\boldsymbol{NH}\boldsymbol{4}}^{\boldsymbol{dz}}$ uptake of NH_4_ by diazotroph (mmol N L^-1^ s^-1^) (Equation S6)

$\boldsymbol{V}_{\boldsymbol{NO}\boldsymbol{3}}^{\boldsymbol{dz}}\boldsymbol{=}\boldsymbol{V}_{\boldsymbol{N,max}}^{\boldsymbol{dz}}\boldsymbol{x}_{\boldsymbol{NO}\boldsymbol{3}}^{\boldsymbol{dz}}$ uptake of NO_3_ by diazotroph (mmol N L^-1^ s^-1^) (Equation S7)

As the diazotroph is facultative, the nitrogen fixation term is equal to the maximum uptake of N multiplied by the dimensionless facultative term ($\boldsymbol{x}_{\boldsymbol{Nfix}}^{\boldsymbol{dz}}$). The facultative term accounts for the fraction of the diazotroph N requirement that is satisfied by the uptake of NO_3_ and NH_4_ and therefore scales nitrogen fixation in order to provide the extra N requirement, permitting that diazotroph growth isn’t limited by P, Fe, T or Light. The facultative term essentially indicates how much nitrogen fixation the diazotroph is performing.

$\boldsymbol{V}_{\boldsymbol{NFIX}}^{\boldsymbol{dz}}\boldsymbol{=}\boldsymbol{V}_{\boldsymbol{N,max}}^{\boldsymbol{dz}}$ uptake of N_2_ through nitrogen fixation (mmol N L^-1^ s^-1^) (Equation S8)

$\boldsymbol{x}_{\boldsymbol{Nfix}}^{\boldsymbol{dz}}\boldsymbol{=1-}\left( \boldsymbol{x}_{\boldsymbol{NO}\boldsymbol{3}}^{\boldsymbol{dz}}\boldsymbol{+}\boldsymbol{x}_{\boldsymbol{NH}\boldsymbol{4}}^{\boldsymbol{dz}} \right)$ Facultative term, how much nitrogen fixation is being

performed (Equation S9)

The uptake of NH_4_ ($\boldsymbol{V}_{\boldsymbol{NH}\boldsymbol{4}}^{\boldsymbol{dz}}$), NO_3_ ($\boldsymbol{V}_{\boldsymbol{NO}\boldsymbol{3}}^{\boldsymbol{dz}}$) and N_2_ ($\boldsymbol{V}_{\boldsymbol{NFIX}}^{\boldsymbol{dz}}$) by the diazotroph require the calculation of the maximum uptake of N ($\boldsymbol{V}_{\boldsymbol{N,max}}^{\boldsymbol{dz}}$) and the proportion of N uptake that is supplied through uptake of NH_4_ ($\boldsymbol{x}_{\boldsymbol{NH}\boldsymbol{4}}^{\boldsymbol{dz}}$) and NO_3_ ($\boldsymbol{x}_{\boldsymbol{NO}\boldsymbol{3}}^{\boldsymbol{dz}}$) based upon how N is allocated between internal enzymes and surface sites ($\boldsymbol{F}_{\boldsymbol{A, N}}^{\boldsymbol{dz}}$). For N allocation to surface sites ($\boldsymbol{F}_{\boldsymbol{A, N}}^{\boldsymbol{dz}}$) both NO_3_ and NH_4_ are accounted for simultaneously.

$\boldsymbol{F}_{\boldsymbol{A, N}}^{\boldsymbol{dz}}\boldsymbol{=}\frac{\boldsymbol{1}}{\boldsymbol{1+}\sqrt{\boldsymbol{max}\left( \frac{\boldsymbol{NO}\boldsymbol{3}}{\boldsymbol{K}_{\boldsymbol{NO}\boldsymbol{3}}^{\boldsymbol{dz}}}\boldsymbol{,}\frac{\boldsymbol{NH}\boldsymbol{4}}{\boldsymbol{K}_{\boldsymbol{NH}\boldsymbol{4}}^{\boldsymbol{dz}}} \right)}}$ Allocation of N to surface sites (Equation S10)

$\boldsymbol{x}_{\boldsymbol{NH}\boldsymbol{4}}^{\boldsymbol{dz}}\boldsymbol{=}\left( \frac{\boldsymbol{NH}\boldsymbol{4}}{{\left( \frac{\left( \boldsymbol{1-}\boldsymbol{F}_{\boldsymbol{A, N}}^{\boldsymbol{dz}} \right)}{\boldsymbol{F}_{\boldsymbol{A,N}}^{\boldsymbol{dz}}} \right)\boldsymbol{K}}_{\boldsymbol{NH}\boldsymbol{4}}^{\boldsymbol{dz}}\boldsymbol{+NH}\boldsymbol{4}} \right)\boldsymbol{\times}\left( \boldsymbol{1-}\boldsymbol{F}_{\boldsymbol{A, N}}^{\boldsymbol{dz}} \right)$ Proportion of N uptake via NH_4_ (Equation S11)

$\boldsymbol{x}_{\boldsymbol{NO}\boldsymbol{3}}^{\boldsymbol{dz}}\boldsymbol{=}\left( \left( \frac{\boldsymbol{NO}\boldsymbol{3}}{{\left( \frac{\left( \boldsymbol{1-}\boldsymbol{F}_{\boldsymbol{A,N}}^{\boldsymbol{dz}} \right)}{\boldsymbol{F}_{\boldsymbol{A,N}}^{\boldsymbol{dz}}} \right)\boldsymbol{K}}_{\boldsymbol{NO}\boldsymbol{3}}^{\boldsymbol{dz}}\boldsymbol{+NO}\boldsymbol{3}} \right)\boldsymbol{\times}\left( \boldsymbol{1-}\left( \frac{\boldsymbol{NH}\boldsymbol{4}}{{\left( \frac{\left( \boldsymbol{1-}\boldsymbol{F}_{\boldsymbol{A,N}}^{\boldsymbol{dz}} \right)}{\boldsymbol{F}_{\boldsymbol{A,N}}^{\boldsymbol{dz}}} \right)\boldsymbol{K}}_{\boldsymbol{NH}\boldsymbol{4}}^{\boldsymbol{dz}}\boldsymbol{+NH}\boldsymbol{4}} \right) \right) \right)\boldsymbol{\times}\left( \boldsymbol{1-}\boldsymbol{F}_{\boldsymbol{A,N}}^{\boldsymbol{dz}} \right)$ Proportion of N uptake via NO_3_

(Equation S12)

$\boldsymbol{NO}\boldsymbol{3}$ = Available nitrate pool (μmol L^-1^)

$\boldsymbol{NH}\boldsymbol{4}$ = Available ammonium pool (μmol L^-1^)

$\boldsymbol{F}_{\boldsymbol{A,N}}^{\boldsymbol{dz}}$ = N allocated to surface sites (unitless)

$\boldsymbol{K}_{\boldsymbol{NH}\boldsymbol{4}}^{\boldsymbol{dz}}$ = 0.13 µmol N L^-1^  Half saturation constant for NH_4_

$\boldsymbol{K}_{\boldsymbol{NO}\boldsymbol{3}}^{\boldsymbol{dz}}$ = 0.39 µmol N L^-1^  Half saturation constant for NO_3_

$\boldsymbol{V}_{\boldsymbol{N,max}}^{\boldsymbol{dz}}\boldsymbol{=}\frac{\boldsymbol{\mu}_{\boldsymbol{max}}^{\boldsymbol{dz}}}{\boldsymbol{\zeta}_{\boldsymbol{N}}}\left( \frac{\boldsymbol{Q}_{\boldsymbol{N,min}}^{\boldsymbol{dz}}}{\boldsymbol{Q}_{\boldsymbol{N}}^{\boldsymbol{dz}}} \right)\left( \boldsymbol{1-\xi}\boldsymbol{Q}_{\boldsymbol{Chl}}^{\boldsymbol{dz,C}} \right)\left( \frac{\boldsymbol{(1-}\boldsymbol{Q}_{\boldsymbol{N}}^{\boldsymbol{dz}}\boldsymbol{/}\boldsymbol{Q}_{\boldsymbol{N,max}}^{\boldsymbol{dz}}\boldsymbol{)}^{\boldsymbol{2}}}{\boldsymbol{(1-}\boldsymbol{Q}_{\boldsymbol{N}}^{\boldsymbol{dz}}\boldsymbol{/}\boldsymbol{Q}_{\boldsymbol{N,max}}^{\boldsymbol{dz}}\boldsymbol{)}^{\boldsymbol{2}}\boldsymbol{+}\boldsymbol{(K}_{\boldsymbol{inh}}\boldsymbol{)}^{\boldsymbol{2}}} \right)\boldsymbol{\times max}\left( \boldsymbol{L}_{\boldsymbol{P}}^{\boldsymbol{dz}}\boldsymbol{,}\boldsymbol{L}_{\boldsymbol{Fe}}^{\boldsymbol{dz}} \right)$ Max uptake rate of N

(Equation S13)

$\boldsymbol{\zeta}_{\boldsymbol{N}}$ = 2.3 mol C mol^-1^ N cost of biosynthesis based on N

$\boldsymbol{Q}_{\boldsymbol{N,min}}^{\boldsymbol{dz}}$ = 0.13 mol N mol^-1^ C Minimum N quota of diazotroph

$\boldsymbol{Q}_{\boldsymbol{N,max}}^{\boldsymbol{dz}}$ = 0.16 mol N mol^-1^ C Maximum N quota of diazotroph

$\boldsymbol{Q}_{\boldsymbol{N}}^{\boldsymbol{dz}}$ = Actual N:C quota of diazotroph (mol/mol)

$\boldsymbol{\xi}\boldsymbol{Q}_{\boldsymbol{Chl}}^{\boldsymbol{dz,C}}$ = fraction of protoplasmic N required for pigment synthesis (unitless)

$\boldsymbol{1-\xi}\boldsymbol{Q}_{\boldsymbol{Chl}}^{\boldsymbol{dz,C}}$= fraction of protoplasmic N remaining for nutrient uptake and assimilation (unitless)

$\boldsymbol{L}_{\boldsymbol{P}}^{\boldsymbol{dz}}\boldsymbol{,}\boldsymbol{L}_{\boldsymbol{Fe}}^{\boldsymbol{dz}}$ = Fraction of Diazotroph maximum growth rate allowed by P and Fe

limitation (unitless)

$\boldsymbol{K}_{\boldsymbol{inh}}$ = 0.05 (unitless)

- 1. **Diazotroph Phosphorus Biomass**

Diazotroph P biomass ($\boldsymbol{P}_{\boldsymbol{P}}^{\boldsymbol{dz}}\boldsymbol{)}$ is parameterised following the other PFTs in the model. For maximum P uptake by diazotrophs however, the uptake of nitrogen via nitrogen fixation is added to account for the extra P requirement of nitrogen fixation.

$$\frac{\boldsymbol{\partial P}_{\boldsymbol{P}}^{\boldsymbol{dz}}}{\boldsymbol{\partial t}}\boldsymbol{=}\left( \boldsymbol{1-}\boldsymbol{\partial}^{\boldsymbol{dz}} \right)\left( \boldsymbol{V}_{\boldsymbol{PO}\boldsymbol{4}}^{\boldsymbol{dz}}\boldsymbol{+}\boldsymbol{V}_{\boldsymbol{DOP}}^{\boldsymbol{dz}} \right)\boldsymbol{P}_{\boldsymbol{C}}^{\boldsymbol{dz}}\boldsymbol{-}\boldsymbol{m}^{\boldsymbol{dz}}\frac{\boldsymbol{P}_{\boldsymbol{C}}^{\boldsymbol{dz}}}{\boldsymbol{K}_{\boldsymbol{m}}\boldsymbol{+}\boldsymbol{P}_{\boldsymbol{C}}^{\boldsymbol{dz}}}\boldsymbol{P}_{\boldsymbol{P}}^{\boldsymbol{dz}}\boldsymbol{-sh}\boldsymbol{\times}\boldsymbol{w}^{\boldsymbol{dz}}\boldsymbol{P}_{\boldsymbol{C}}^{\boldsymbol{dz}}\boldsymbol{P}_{\boldsymbol{P}}^{\boldsymbol{dz}}\boldsymbol{-}\boldsymbol{g}^{\boldsymbol{Z}}\left( \boldsymbol{P}_{\boldsymbol{C}}^{\boldsymbol{dz}} \right)\boldsymbol{Z}\boldsymbol{Q}_{\boldsymbol{P}}^{\boldsymbol{dz}}\boldsymbol{-}\boldsymbol{g}^{\boldsymbol{M}}\left( \boldsymbol{P}_{\boldsymbol{C}}^{\boldsymbol{dz}} \right)\boldsymbol{M}\boldsymbol{Q}_{\boldsymbol{P}}^{\boldsymbol{dz}}$$

(Equation S14)

Uptake of P:

$\boldsymbol{V}_{\boldsymbol{PO}\boldsymbol{4}}^{\boldsymbol{dz}}\boldsymbol{=}\boldsymbol{V}_{\boldsymbol{P,max}}^{\boldsymbol{dz}}\left( \frac{\boldsymbol{PO}\boldsymbol{4}}{{\left( \frac{\left( \boldsymbol{1-}\boldsymbol{F}_{\boldsymbol{A,P}}^{\boldsymbol{dz}} \right)}{\boldsymbol{F}_{\boldsymbol{A,P}}^{\boldsymbol{dz}}} \right)\boldsymbol{K}}_{\boldsymbol{PO}\boldsymbol{4}}^{\boldsymbol{dz}}\boldsymbol{+PO}\boldsymbol{4}} \right)\boldsymbol{\times}\left( \boldsymbol{1-}\boldsymbol{F}_{\boldsymbol{A,P}}^{\boldsymbol{dz}} \right)$

uptake of PO_4_ by diazotroph (mmol P L^-1^ s^-1^)

(Equation S15)

$\boldsymbol{V}_{\boldsymbol{DOP}}^{\boldsymbol{dz}}\boldsymbol{=}\boldsymbol{V}_{\boldsymbol{P,max}}^{\boldsymbol{dz}}\left( \frac{\boldsymbol{DOP}}{\boldsymbol{K}_{\boldsymbol{DOP}}^{\boldsymbol{dz}}\boldsymbol{+DOP}}\boldsymbol{\times}\left( \boldsymbol{1-}\left( \frac{\boldsymbol{PO}\boldsymbol{4}}{{\left( \frac{\left( \boldsymbol{1-}\boldsymbol{F}_{\boldsymbol{A,P}}^{\boldsymbol{dz}} \right)}{\boldsymbol{F}_{\boldsymbol{A,P}}^{\boldsymbol{dz}}} \right)\boldsymbol{K}}_{\boldsymbol{PO}\boldsymbol{4}}^{\boldsymbol{dz}}\boldsymbol{+PO}\boldsymbol{4}} \right) \right) \right)$

DOP uptake by diazotroph (mmol P L^-1^ s^-1^)

(Equation S16)

$\boldsymbol{PO}\boldsymbol{4}$ = Available phosphate pool (μmol L^-1^)

$\boldsymbol{DOP}$ = Available DOP pool (μmol L^-1^)

$\boldsymbol{F}_{\boldsymbol{A,P}}^{\boldsymbol{dz}}$ = P allocated to surface sites (unitless)

$\boldsymbol{K}_{\boldsymbol{PO}\boldsymbol{4}}^{\boldsymbol{dz}}$ = 0.012 µmol P L^-1^ (nanophytoplankton = 0.024 µmol P L^-1^)

Half saturation constant for PO_4_

$\boldsymbol{K}_{\boldsymbol{DOP}}^{\boldsymbol{dz}}$ = 3.4 µmol P L^-1^  Half saturation constant for DOP

$\boldsymbol{V}_{\boldsymbol{P,max}}^{\boldsymbol{dz}}\boldsymbol{=}\left( \frac{\boldsymbol{P}_{\boldsymbol{0}}}{\boldsymbol{N}_{\boldsymbol{0}}} \right)\frac{\boldsymbol{\mu}_{\boldsymbol{max}}^{\boldsymbol{dz}}}{\boldsymbol{\zeta}_{\boldsymbol{N}}}\left( \frac{\boldsymbol{Q}_{\boldsymbol{N,min}}^{\boldsymbol{dz}}}{\boldsymbol{Q}_{\boldsymbol{N}}^{\boldsymbol{dz}}} \right)\left( \boldsymbol{1-\xi}\boldsymbol{Q}_{\boldsymbol{Chl}}^{\boldsymbol{dz,C}} \right)\boldsymbol{L}_{\boldsymbol{Fe}}^{\boldsymbol{dz}}\left( \frac{\boldsymbol{(1-}\boldsymbol{Q}_{\boldsymbol{P}}^{\boldsymbol{dz}}\boldsymbol{/}\boldsymbol{Q}_{\boldsymbol{P,max}}^{\boldsymbol{dz}}\boldsymbol{)}^{\boldsymbol{2}}}{\boldsymbol{(1-}\boldsymbol{Q}_{\boldsymbol{P}}^{\boldsymbol{dz}}\boldsymbol{/}\boldsymbol{Q}_{\boldsymbol{P,max}}^{\boldsymbol{dz}}\boldsymbol{)}^{\boldsymbol{2}}\boldsymbol{+}\boldsymbol{(K}_{\boldsymbol{inh}}\boldsymbol{)}^{\boldsymbol{2}}} \right)\boldsymbol{+}\boldsymbol{V}_{\boldsymbol{Nfix}}^{\boldsymbol{dz}}$ Max uptake rate of P

(Equation S17)

$\boldsymbol{Q}_{\boldsymbol{P,max}}^{\boldsymbol{dz}}$ = 11 mmol P mol^-1^ C Maximum P quota of diazotroph

$\boldsymbol{Q}_{\boldsymbol{P}}^{\boldsymbol{dz}}$ = Actual P:C quota of diazotroph (mol/mol)

- 1. **Diazotroph Iron Biomass**

Diazotroph Fe biomass is parameterised in the same way as the other PFTs however, there is an additional Fe cost associated with nitrogen fixation which is included in the calculation of Fe limitation (see section 2 of main text).

$$\frac{\boldsymbol{\partial P}_{\boldsymbol{Fe}}^{\boldsymbol{dz}}}{\boldsymbol{\partial t}}\boldsymbol{=}\left( \boldsymbol{1-}\boldsymbol{\partial}^{\boldsymbol{dz}} \right)\boldsymbol{V}_{\boldsymbol{Fe}}^{\boldsymbol{dz}}\boldsymbol{P}_{\boldsymbol{C}}^{\boldsymbol{dz}}\boldsymbol{-}\boldsymbol{m}^{\boldsymbol{dz}}\frac{\boldsymbol{P}_{\boldsymbol{C}}^{\boldsymbol{dz}}}{\boldsymbol{k}_{\boldsymbol{m}}\boldsymbol{+}\boldsymbol{P}_{\boldsymbol{C}}^{\boldsymbol{dz}}}\boldsymbol{P}_{\boldsymbol{Fe}}^{\boldsymbol{dz}}\boldsymbol{-sh}\boldsymbol{\times}\boldsymbol{w}^{\boldsymbol{dz}}\boldsymbol{P}_{\boldsymbol{C}}^{\boldsymbol{dz}}\boldsymbol{P}_{\boldsymbol{Fe}}^{\boldsymbol{dz}}\boldsymbol{-}\boldsymbol{g}^{\boldsymbol{Z}}\left( \boldsymbol{P}^{\boldsymbol{dz}} \right)\boldsymbol{Z}\boldsymbol{Q}_{\boldsymbol{Fe}}^{\boldsymbol{dz}}\boldsymbol{-}\boldsymbol{g}^{\boldsymbol{M}}\left( \boldsymbol{P}^{\boldsymbol{dz}} \right)\boldsymbol{M}\boldsymbol{Q}_{\boldsymbol{Fe}}^{\boldsymbol{dz}}$$

(Equation S18)

Uptake of Fe:

$\boldsymbol{V}_{\boldsymbol{Fe}}^{\boldsymbol{dz}}\boldsymbol{=}\boldsymbol{V}_{\boldsymbol{Fe,max}}^{\boldsymbol{dz}}\boldsymbol{x}_{\boldsymbol{Fe}}^{\boldsymbol{dz}}\left( \frac{\boldsymbol{1.8\times}\boldsymbol{x}_{\boldsymbol{NO}\boldsymbol{3}}^{\boldsymbol{dz}}}{\boldsymbol{x}_{\boldsymbol{NO}\boldsymbol{3}}^{\boldsymbol{dz}}\boldsymbol{+}\boldsymbol{x}_{\boldsymbol{NH}\boldsymbol{4}}^{\boldsymbol{dz}}}\boldsymbol{\times}\left( \boldsymbol{1-}\boldsymbol{x}_{\boldsymbol{Fe}}^{\boldsymbol{dz}} \right) \right)$ Uptake of Fe by diazotroph (Equation S19)

$\boldsymbol{V}_{\boldsymbol{Fe,max}}^{\boldsymbol{dz}}\boldsymbol{=}\boldsymbol{Q}_{\boldsymbol{Fe,max}}^{\boldsymbol{dz}}\frac{\boldsymbol{\mu}_{\boldsymbol{max}}^{\boldsymbol{dz}}}{\boldsymbol{\zeta}_{\boldsymbol{N}}}\left( \frac{\boldsymbol{Q}_{\boldsymbol{N,min}}^{\boldsymbol{dz}}}{\boldsymbol{Q}_{\boldsymbol{N}}^{\boldsymbol{dz}}} \right)\left( \boldsymbol{1-\xi}\boldsymbol{Q}_{\boldsymbol{Chl}}^{\boldsymbol{dz,C}} \right)\left( \frac{\boldsymbol{(1-}\boldsymbol{Q}_{\boldsymbol{Fe}}^{\boldsymbol{dz}}\boldsymbol{/}\boldsymbol{Q}_{\boldsymbol{Fe,max}}^{\boldsymbol{dz}}\boldsymbol{)}^{\boldsymbol{2}}}{\boldsymbol{(1-}\boldsymbol{Q}_{\boldsymbol{Fe}}^{\boldsymbol{dz}}\boldsymbol{/}\boldsymbol{Q}_{\boldsymbol{Fe,max}}^{\boldsymbol{dz}}\boldsymbol{)}^{\boldsymbol{2}}\boldsymbol{+}\boldsymbol{(K}_{\boldsymbol{inh}}\boldsymbol{)}^{\boldsymbol{2}}} \right)$

Max uptake rate of Fe

(Equation S20)

$\boldsymbol{x}_{\boldsymbol{Fe}}^{\boldsymbol{dz}}\boldsymbol{=}\left( \frac{\boldsymbol{Fe}}{{\left( \frac{\left( \boldsymbol{1-}\boldsymbol{F}_{\boldsymbol{A,Fe}}^{\boldsymbol{dz}} \right)}{\boldsymbol{F}_{\boldsymbol{A,Fe}}^{\boldsymbol{dz}}} \right)\boldsymbol{K}}_{\boldsymbol{Fe}}^{\boldsymbol{dz}}\boldsymbol{+Fe}} \right)\boldsymbol{\times}\left( \boldsymbol{1-}\boldsymbol{F}_{\boldsymbol{A,Fe}}^{\boldsymbol{dz}} \right)$ Cellular allocation of Fe (Equation S21)

$\boldsymbol{Q}_{\boldsymbol{Fe,max}}^{\boldsymbol{dz}}$ = 80x10^-6^ mol Fe mol^-1^ C Maximum Fe quota of diazotroph

$\boldsymbol{Q}_{\boldsymbol{Fe}}^{\boldsymbol{dz}}$ = Actual Fe:C quota of diazotroph (mol/mol)

$\boldsymbol{K}_{\boldsymbol{Fe}}^{\boldsymbol{dz}}$ = 10 nmol Fe L^-1^ (nanophytoplankton = 3 nmol Fe L^-1^) Half saturation constant for Fe

$\boldsymbol{F}_{\boldsymbol{A,Fe}}^{\boldsymbol{dz}}$ = Fe allocated to surface sites (unitless)

- 1. **Diazotroph Chlorophyll Biomass**

Chlorophyll biomass ($\boldsymbol{P}_{\boldsymbol{Chl}}^{\boldsymbol{dz}}$**)** is calculated in the same way as the other PFTs in the model using Geider et al. (1998) photoadaptive model which converts nitrogen uptake into assimilated chlorophyll biomass using the ratio of energy assimilated to energy absorbed ($\boldsymbol{\rho}^{\boldsymbol{dz,Chl}}\boldsymbol{,}$ equation S23). The value of 144 in equation S23 is the square of the molar mass of C and is used to convert from mol to mg as chlorophyll is reported in mg Chl m^-3^.

$$\frac{\boldsymbol{\partial P}_{\boldsymbol{Chl}}^{\boldsymbol{dz}}}{\boldsymbol{\partial t}}\boldsymbol{=}\left( \boldsymbol{1-}\boldsymbol{\partial}^{\boldsymbol{dz}} \right)\boldsymbol{Q}_{\boldsymbol{Chl,max}}^{\boldsymbol{dz,N}}\boldsymbol{\times}\boldsymbol{\rho}^{\boldsymbol{dz,Chl}}\left( \boldsymbol{V}_{\boldsymbol{NO}\boldsymbol{3}}^{\boldsymbol{dz}}\boldsymbol{+}\boldsymbol{V}_{\boldsymbol{NH}\boldsymbol{4}}^{\boldsymbol{dz}}\boldsymbol{+}\boldsymbol{V}_{\boldsymbol{NFIX}}^{\boldsymbol{dz}} \right)\boldsymbol{P}_{\boldsymbol{C}}^{\boldsymbol{dz}}\boldsymbol{-}\boldsymbol{m}^{\boldsymbol{dz}}\frac{\boldsymbol{P}_{\boldsymbol{C}}^{\boldsymbol{dz}}}{\boldsymbol{k}_{\boldsymbol{m}}\boldsymbol{+}\boldsymbol{P}_{\boldsymbol{C}}^{\boldsymbol{dz}}}\boldsymbol{P}_{\boldsymbol{Chl}}^{\boldsymbol{dz}}\boldsymbol{-sh}\boldsymbol{\times}\boldsymbol{w}^{\boldsymbol{dz}}\boldsymbol{P}_{\boldsymbol{C}}^{\boldsymbol{dz}}\boldsymbol{P}_{\boldsymbol{Chl}}^{\boldsymbol{dz}}\boldsymbol{-}\boldsymbol{g}^{\boldsymbol{Z}}\left( \boldsymbol{P}^{\boldsymbol{dz}} \right)\boldsymbol{Z}\boldsymbol{Q}_{\boldsymbol{Chl}}^{\boldsymbol{dz,C}}\boldsymbol{-}\boldsymbol{g}^{\boldsymbol{M}}\left( \boldsymbol{P}^{\boldsymbol{dz}} \right)\boldsymbol{M}\boldsymbol{Q}_{\boldsymbol{Chl}}^{\boldsymbol{dz,C}}$$

(Equation S22)

$\boldsymbol{\rho}^{\boldsymbol{dz,Chl}}\boldsymbol{=}\frac{144\boldsymbol{\mu}_{\boldsymbol{P}}^{\boldsymbol{dz}}\boldsymbol{P}_{\boldsymbol{C}}^{\boldsymbol{dz}}}{\boldsymbol{\alpha}^{\boldsymbol{dz}}\boldsymbol{P}_{\boldsymbol{Chl}}^{\boldsymbol{dz}}\boldsymbol{PAR}^{\boldsymbol{dz}}}$ energy assimilated: energy absorbed (Geider et al., 1996)

(Equation S23)

$\boldsymbol{Q}_{\boldsymbol{Chl,max}}^{\boldsymbol{dz,N}}$ = 0.3 g Chl g^-1^ N Maximum Chl to N ratio of diazotroph

$\boldsymbol{Q}_{\boldsymbol{Chl}}^{\boldsymbol{dz,C}}$ = Actual Chl:C ratio of the diazotroph (g Chl g^-1^ C)

$\boldsymbol{PAR}^{\boldsymbol{dz}}$ = Photosynthetically available radiation (W m^-2^)

$\boldsymbol{\alpha}^{\boldsymbol{dz}}$ = 2 Wm^-2^ d^-1^ (nanophytoplankton = 4 Wm^-2^ d^-1^) Initial slope of P-I curve

- 1. **Nutrient Limitation**

The nutrient limitation terms are calculated in the same way for all phytoplankton. Nutrient limitation within PISCES QUOTA is controlled by the optimal allocation of resources and a chain of limitations such that the cellular P controls N assimilation (Pahlow 2005, Pahlow et al., 2013, Kwiatkowski et al., 2018, Pahlow et al., 2020). Therefore, overall nutrient limitation only considers N and Fe limitation as P limitation is accounted for within N limitation (Equation S24).

$\boldsymbol{L}_{\boldsymbol{lim}}^{\boldsymbol{dz}}\boldsymbol{=min(}\boldsymbol{L}_{\boldsymbol{N}}^{\boldsymbol{dz}}\boldsymbol{,}\boldsymbol{L}_{\boldsymbol{Fe}}^{\boldsymbol{dz}}\boldsymbol{)}$ Overall nutrient limitation (Equation S24)

$\boldsymbol{L}_{\boldsymbol{N}}^{\boldsymbol{dz}}\boldsymbol{=max}\left( \boldsymbol{0,}\frac{\boldsymbol{1-}\boldsymbol{Q}_{\boldsymbol{N,min}}^{\boldsymbol{dz}}\boldsymbol{/}\boldsymbol{Q}_{\boldsymbol{N}}^{\boldsymbol{dz}}}{\boldsymbol{1-}\boldsymbol{Q}_{\boldsymbol{N,min}}^{\boldsymbol{dz}}\boldsymbol{/}\boldsymbol{Q}_{\boldsymbol{N,max}}^{\boldsymbol{dz}}} \right)$ N limitation (Equation S25)

$\boldsymbol{Q}_{\boldsymbol{N,min}}^{\boldsymbol{dz}}$ = 0.13 mol N mol^-1^ C (nanophytoplankton = 0.08 mol N mol^-1^ C)

Minimum N quota of diazotroph

$\boldsymbol{Q}_{\boldsymbol{N,max}}^{\boldsymbol{dz}}$ = 0.16 mol N mol^-1^ C Maximum N quota of diazotroph

$\boldsymbol{Q}_{\boldsymbol{N}}^{\boldsymbol{dz}}$ = Actual N:C quota of diazotroph (mol/mol)

$\boldsymbol{L}_{\boldsymbol{P}}^{\boldsymbol{dz}}\boldsymbol{=min}\left( \boldsymbol{1,}\frac{\boldsymbol{Q}_{\boldsymbol{P}}^{\boldsymbol{dz}}\boldsymbol{-}\boldsymbol{Q}_{\boldsymbol{P,min}}^{\boldsymbol{dz}}}{\boldsymbol{Q}_{\boldsymbol{P,max}}^{\boldsymbol{dz}}\boldsymbol{-}\boldsymbol{Q}_{\boldsymbol{P,min}}^{\boldsymbol{dz}}} \right)$ P limitation (Equation S26)

$\boldsymbol{Q}_{\boldsymbol{P,min}}^{\boldsymbol{dz}}$ = 2.8 mmol P mol^-1^ C (nanophytoplankton = 2.2 mmol P mol^-1^ C)

Minimum P quota of diazotroph

$\boldsymbol{Q}_{\boldsymbol{P,max}}^{\boldsymbol{dz}}$ = 11 mmol P mol^-1^ C Maximum P quota of diazotroph

$\boldsymbol{Q}_{\boldsymbol{P}}^{\boldsymbol{dz}}$ = Actual P:C quota of diazotroph (mol/mol)

$\boldsymbol{L}_{\boldsymbol{Fe}}^{\boldsymbol{dz}}\boldsymbol{=max}\left( \boldsymbol{0,}\frac{\boldsymbol{Q}_{\boldsymbol{Fe}}^{\boldsymbol{dz}}\boldsymbol{-}\boldsymbol{Q}_{\boldsymbol{Fe,min}}^{\boldsymbol{dz}}}{\boldsymbol{Q}_{\boldsymbol{Fe,opt}}^{\boldsymbol{dz}}} \right)$ Fe limitation (Equation S27)

$\boldsymbol{Q}_{\boldsymbol{Fe,opt}}^{\boldsymbol{dz}}$ = 21x10^-6^ mol Fe mol^-1^ C (nanophytoplankton = 7x10^-6^ mol Fe mol^-1^ C)

Optimum Fe quota of diazotroph

$\boldsymbol{Q}_{\boldsymbol{Fe}}^{\boldsymbol{dz}}$ = Actual Fe:C quota of diazotroph (mol/mol)

The calculation of diazotroph maximum growth rate ($\boldsymbol{\mu}_{\boldsymbol{max}}^{\boldsymbol{dz}}$), elemental use efficiencies (EUE) and minimum Fe quota ($Q_{Fe,min}^{dz}$) for both *Trichodesmium* and *Crocosphaera* are included in the main text. A summary of the parameters and their values is provided in supplementary table 2.

1. **Supplementary Tables**

**Table S1.** Area of the ocean that *Trichodesmium* and *Crocosphaera* have been thermally excluded from. Showing both the monthly maximum temperature and annual mean temperature for the historical period (1996-2005) and for several RCP climate forcing scenarios (RCP4.5, RCP6.0 and RCP8.5 (2091- 2100))

|  | **Hist (1996-2005)** | | **RCP4.5 (2091-2100)** | | **RCP6.0 (2091-2100)** | | **RCP8.5 (2091-2100)** | |
| --- | --- | --- | --- | --- | --- | --- | --- | --- |
|  | **Area of diazotroph thermal exclusion** | | **Area of diazotroph thermal exclusion** | | **Area of diazotroph thermal exclusion** | | **Area of diazotroph thermal exclusion** | |
|  | **km^2^** | **%** | **km^2^** | **%** | **km^2^** | **%** | **km^2^** | **%** |
| **Monthly Maximum SST** |  |  |  |  |  |  |  |  |
| Tricho TEUE | 2.91E+08 | 31.5 | 2.61E+08 | 28.2 | 2.50E+08 | 27.0 | 2.19E+08 | 23.7 |
| Croco TEUE | 3.42E+08 | 36.9 | 3.05E+08 | 33.0 | 2.96E+08 | 32.0 | 2.58E+08 | 27.8 |
| **Annual Mean SST** |  |  |  |  |  |  |  |  |
| Tricho TEUE | 3.74E+08 | 40.4 | 3.38E+08 | 36.5 | 3.32E+08 | 35.9 | 3.00E+08 | 32.4 |
| Croco TEUE | 4.54E+08 | 49.0 | 4.06E+08 | 43.9 | 3.97E+08 | 42.8 | 3.56E+08 | 38.5 |

**Table S2.** Table of diazotroph model parameters

| **Parameter** | **Value** | | **Description** | **Reference** |
| --- | --- | --- | --- | --- |
| $\boldsymbol{\zeta}_{\boldsymbol{NO}\boldsymbol{3}}^{\boldsymbol{dz}}$ | 2.3 | mol C mol^-1^ N | **Cost of biosynthesis based on NO_3._ Same as for other PFTs** | **Pahlow (2005)** |
| $\boldsymbol{\zeta}_{\boldsymbol{NH}\boldsymbol{4}}^{\boldsymbol{dz}}$ | 1.8 | mol C mol^-1^ N | **Cost of biosynthesis based on NH_4._ Same as for other PFTs** | **Pahlow (2005)** |
| $\boldsymbol{\zeta}_{\boldsymbol{NFIX}}^{\boldsymbol{dz}}$ | 6.6 | mol C mol^-1^ N | **Cost of biosynthesis based on N_2_.** | **Pahlow et al. (2013)** |
| $\boldsymbol{K}_{\boldsymbol{NO}\boldsymbol{3}}^{\boldsymbol{dz}}$ | 0.39 | µmol N L^-1^ | **NO_3_ half saturation for diazotrophs, same as nano** | **Kwiatkowski et al. (2018)** |
| $\boldsymbol{K}_{\boldsymbol{NH}\boldsymbol{4}}^{\boldsymbol{dz}}$ | 0.13 | µmol N L^-1^ | **NH_4_ half saturation for diazotrophs, same as nano** | **Kwiatkowski et al. (2018)** |
| $\boldsymbol{K}_{\boldsymbol{PO}\boldsymbol{4}}^{\boldsymbol{dz}}$ | 0.012 | µmol P L^-1^ | **PO_4_ half saturation for diazotrophs (1/2x nano)** | ***this study*** |
| $\boldsymbol{K}_{\boldsymbol{DOP}}^{\boldsymbol{dz}}$ | 3.5 | µmol P L^-1^ | **DOP half saturation for diazotrophs** | ***this study*** |
| $\boldsymbol{K}_{\boldsymbol{Fe}}^{\boldsymbol{dz}}$ | 10 | nmol Fe L^-1^ | **Fe half saturation for diazotrophs (~3x nano kFe)** | **Kustka et al. (2003b)** |
| $\boldsymbol{Q}_{\boldsymbol{N,min}}^{\boldsymbol{dz}}$ | 0.13 | mol N mol^-1^ C | **Minimum N quota, increased from nanos following Pahlow et al. (2020)** | **Pahlow et al. (2013)** |
| $\boldsymbol{Q}_{\boldsymbol{N,max}}^{\boldsymbol{dz}}$ | 0.16 | mol N mol^-1^ C | **Maximum N quota, same as nanos** | **Kwiatkowski et al. (2018)** |
| $\boldsymbol{Q}_{\boldsymbol{P,min}}^{\boldsymbol{dz}}$ | 2.8 | mmol P mol^-1^ C | **Minimum P quota, increased from nanos following Pahlow et al. (2020)** | **Pahlow et al. (2013)** |
| $\boldsymbol{Q}_{\boldsymbol{P,max}}^{\boldsymbol{dz}}$ | 11 | mmol P mol^-1^ C | **Maximum P quota, same as nanos** | **Kwiatkowski et al. (2018)** |
| $\boldsymbol{Q}_{\boldsymbol{Fe,opt}}^{\boldsymbol{dz}}$ | 21 | µmol Fe mol^-1^ C | **Optimum Fe quota (3x nanos)** | **Kustka et al. (2003b)** |
| $\boldsymbol{Q}_{\boldsymbol{Fe,max}}^{\boldsymbol{dz}}$ | 80 | µmol Fe mol^-1^ C | **Maximum Fe quota, same as nanos** | **Kwiatkowski et al. (2018)** |
| $\boldsymbol{Q}_{\boldsymbol{KustkaFe,NFIX}}^{\boldsymbol{dz}}$ | 13 | µmol Fe mol^-1^ C | **Fe quota required for nitrogen fixation** | **Kustka et al. (2003a)** |
| $\boldsymbol{Q}_{\boldsymbol{Chl,max}}^{\boldsymbol{dz,N}}$ | 0.3 | mol Chl mol^-1^ N | **Max Chl/N quota, same as nanos** | **Kwiatkowski et al. (2018)** |
| $\boldsymbol{\alpha}^{\boldsymbol{dz}}$ | 2 | Wm^-2^ d^-1^ | **P-I slope, (1/2x nanos) following Pahlow et al. (2013)** | **Pahlow et al. (2013)** |
| **m^dz^** | 0.01 | d^-1^ | **Mortality rate, same as nanos** | **Kwiatkowski et al. (2018)** |
| **w^dz^** | 0.01 | d^-1^ mol^-1^ C | **Quadratic mortality rate, same as nanos** | **Kwiatkowski et al. (2018)** |
| $\boldsymbol{\partial}^{\boldsymbol{dz}}$ | 0.05 |  | **Exudation of DOC, same as nanos** | **Kwiatkowski et al. (2018)** |
| $\boldsymbol{Pref}_{\boldsymbol{meso}}^{\boldsymbol{dz}}$ | 0.2 | **(0.3, 1)** | **Mesozooplankton preference for Diazos, reduced to account for toxicity of diazotrophs. Preference of nanophytoplankton and diatoms shown in parenthesises.** | **Carpenter and Capone (2008)** |
| $\boldsymbol{Pref}_{\boldsymbol{micro}}^{\boldsymbol{dz}}$ | 0.4 | **(1, 0.8, 1)** | **Microzooplankton preference for Diazos, reduced to account for toxicity of diazotrophs. Preference of nanophytoplankton, diatoms and picophytoplankton shown in parenthesises.** | **Carpenter and Capone (2008)** |

1. **Supplementary Figures**


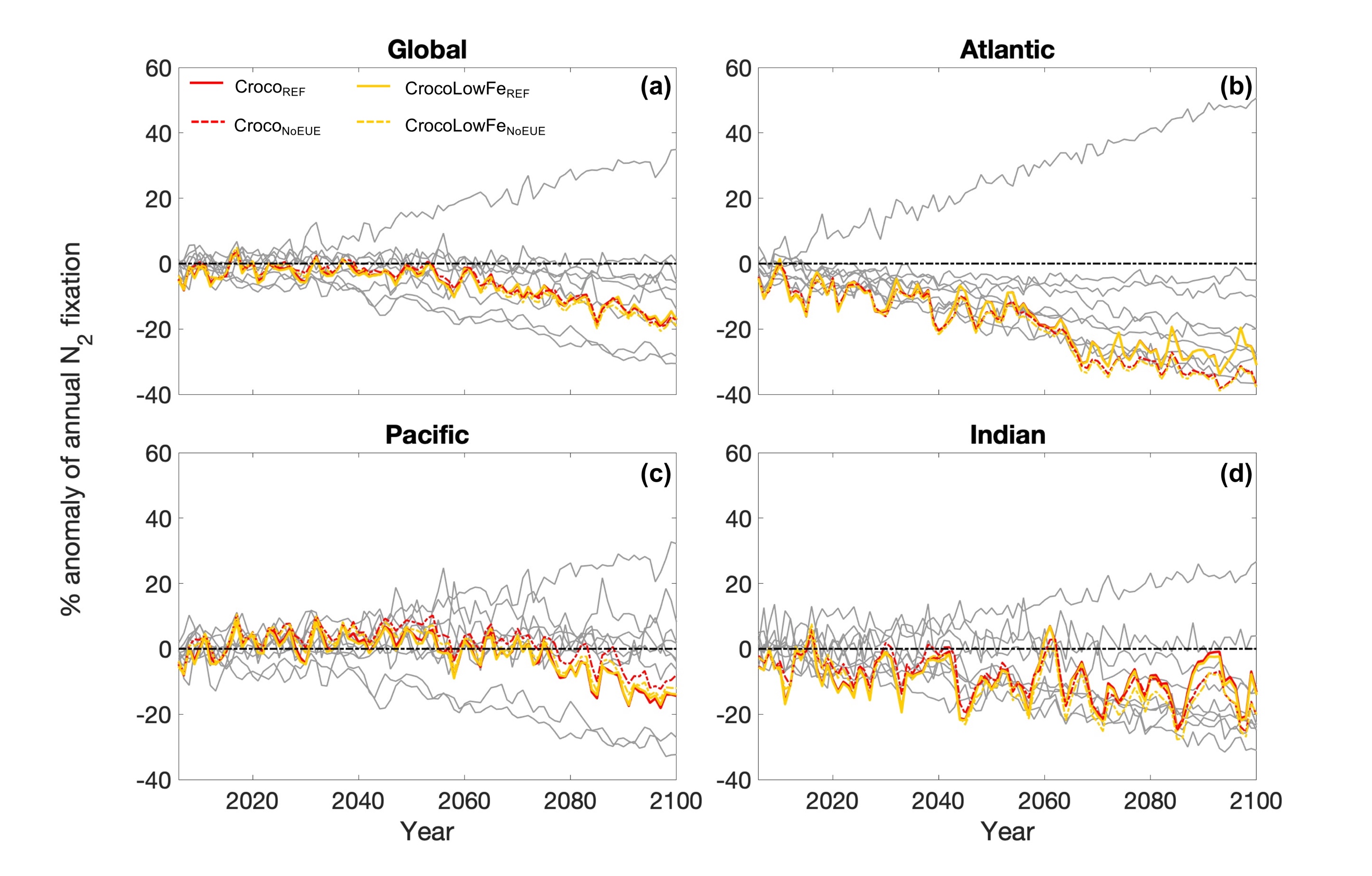


**Fig S1.** Percentage change of integrated nitrogen fixation for the RCP8.5 scenario (2006 – 2100) compared to the historical mean (1996-2005) for Crocosphaera (red) and Crocosphaera with lower Fe cost of N_2_ fixation (yellow), solid lines represent reference simulation where temperature dependent EUEs were included, and dashed lines represent model without EUEs. Percentage change is shown for the global ocean (a) and the ocean basins: (b) Atlantic, (c) Pacific and (d) Indian oceans. Grey lines represent 9 Earth System Models that have been used for climate change projections of nitrogen fixation (Wrightson and Tagliabue, 2020).


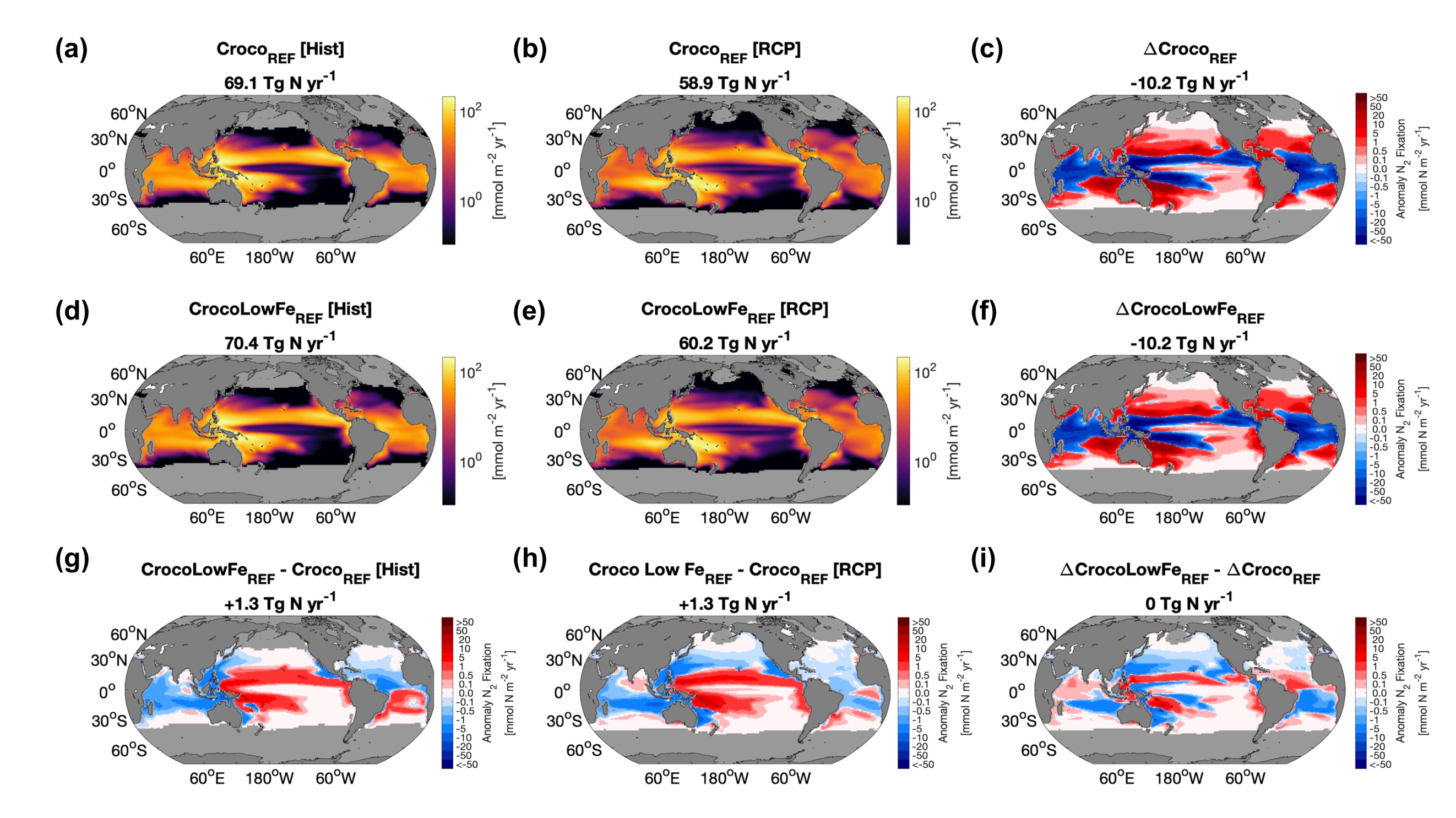


**Fig. S2.** Depth integrated nitrogen fixation for *Crocosphaera* (a, b and c) and *Crocosphaera* with a lower Fe cost of N_2_ fixation (d, e and f) for the historical period (1996-2005; a and d) and the RCP8.5 scenario (2091-2100; b and e). The anomalies between *Crocosphaera* and *Crocosphaera* low Fe cost for the historical period (1996-2005; g) and the RCP8.5 scenario (2091-2100; h) are shown. Also shown are the climate change signal of nitrogen fixation (RCP – historical; c and f) and the anomaly of the climate change signals comparing *Crocosphaera* and *Crocosphaera* low Fe cost (i). Values above the maps indicate globally integrated values of nitrogen fixation (a, b, d and e) and the global anomaly of nitrogen fixation (c, f, g, h and i).


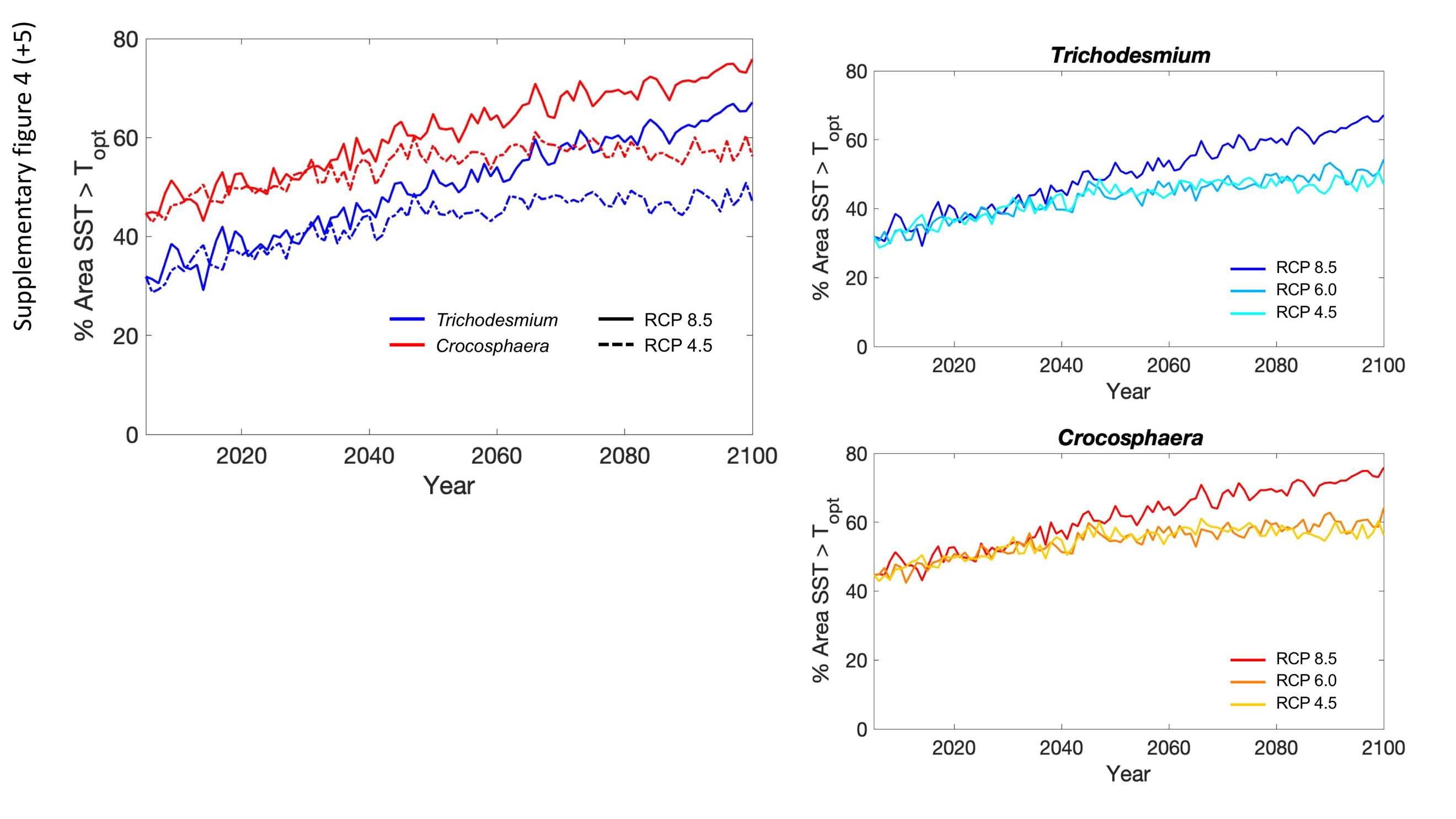


**Fig. S3.** Percentage of the area of the diazotroph’s niche where temperature has surpassed the thermal optimum for growth for both *Trichodesmium* (blue) and *Crocosphaera* (red) using the monthly maximum SST under 2 emission scenarios RCP4.5 (Low, dashed line) and RCP8.5 (high, solid)
